# Synthesis and Characterization of a Terminal Iron(II)–PH_2_ Complex and a Series of Iron(II)–PH_3_ Complexes

**DOI:** 10.1021/acs.inorgchem.4c00605

**Published:** 2024-04-02

**Authors:** Samantha Lau, Mary F. Mahon, Ruth L. Webster

**Affiliations:** † Department of Chemistry, University of Bath, Claverton Down, Bath BA2 7AY, U.K.; ‡ Yusuf Hamied Department of Chemistry, University of Cambridge, Lensfield Road, Cambridge CB2 1EW, U.K.

## Abstract

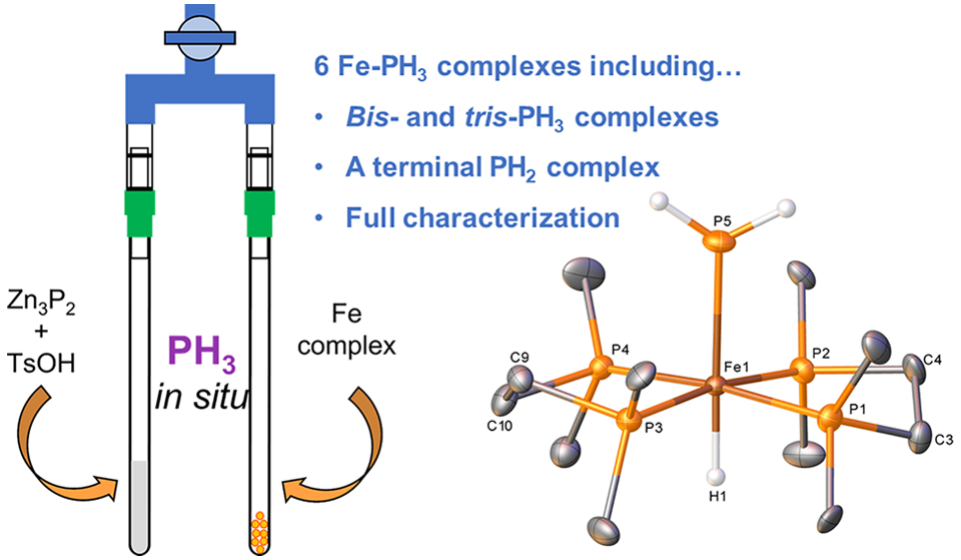

Reported is the reaction of a series of iron(II) bisphosphine complexes with PH_3_ in the presence of NaBAr^F^_4_ [where BAr^F^_4_ = tetrakis(3,5-bis(trifluoromethyl)phenyl)borate]. The iron(II) bisphosphine reagents bear two chlorides or a hydride and a chloride motif. We have isolated six different cationic terminal-bound PH_3_ complexes and undertaken rigorous characterization by NMR spectroscopy, single crystal X-ray diffraction, and mass spectrometry, where the PH_3_ often remains intact during the ionization process. Unusual bis- and tris-PH_3_ complexes are among the compounds isolated. Changing the monophosphine from PH_3_ to PMe_3_ results in the formation of an unusual Fe_7_ cluster, but with no PMe_3_ being ligated. Finally, by using an iron(0) source, we have provided a rare example of a terminally bound iron–PH_2_ complex.

## Abstract 2

Presented is a procedurally simple method for the release of small quantities of PH_3_ gas under inert atmosphere conditions. We have employed this method for the preparation of a series of PH_3_-ligated iron(II) bisphosphine complexes, along with an example of a terminal iron–phosphide complex, which is formed by oxidative addition to Fe(0). When PH_3_ is swapped for PMe_3,_ it is clear that sterics play a role in the coordination chemistry.

## Introduction

Bisphosphine ligands are ubiquitous in catalysis and coordination chemistry. Iron bisphosphine complexes have been of interest to several research groups because of their propensity to catalyze C–C bond-forming reactions and there is a wealth of information that can be gained through simple ligand modification.^1−9^ Synthesis of simple iron bisphosphine complexes featuring ligands such as 1,1-bis(diphenylphopshino)methane (dppm),^10−12^ 1,1-bis(diphenylphosphino)ethane (dppe),^13,14^ and 1,2-bis(dimethylphosphino)ethane (dmpe)^15^ are a good starting point from which to study fundamental reactivity. For example, iron complexes chelated by dmpe have been studied extensively in the activation of CO_2_^16−19^ and N_2_,^20−23^ along with X–H bond activation (X = H,^24^ B,^25^ C,^26^ and N^27,28^).

**Figure 1:**
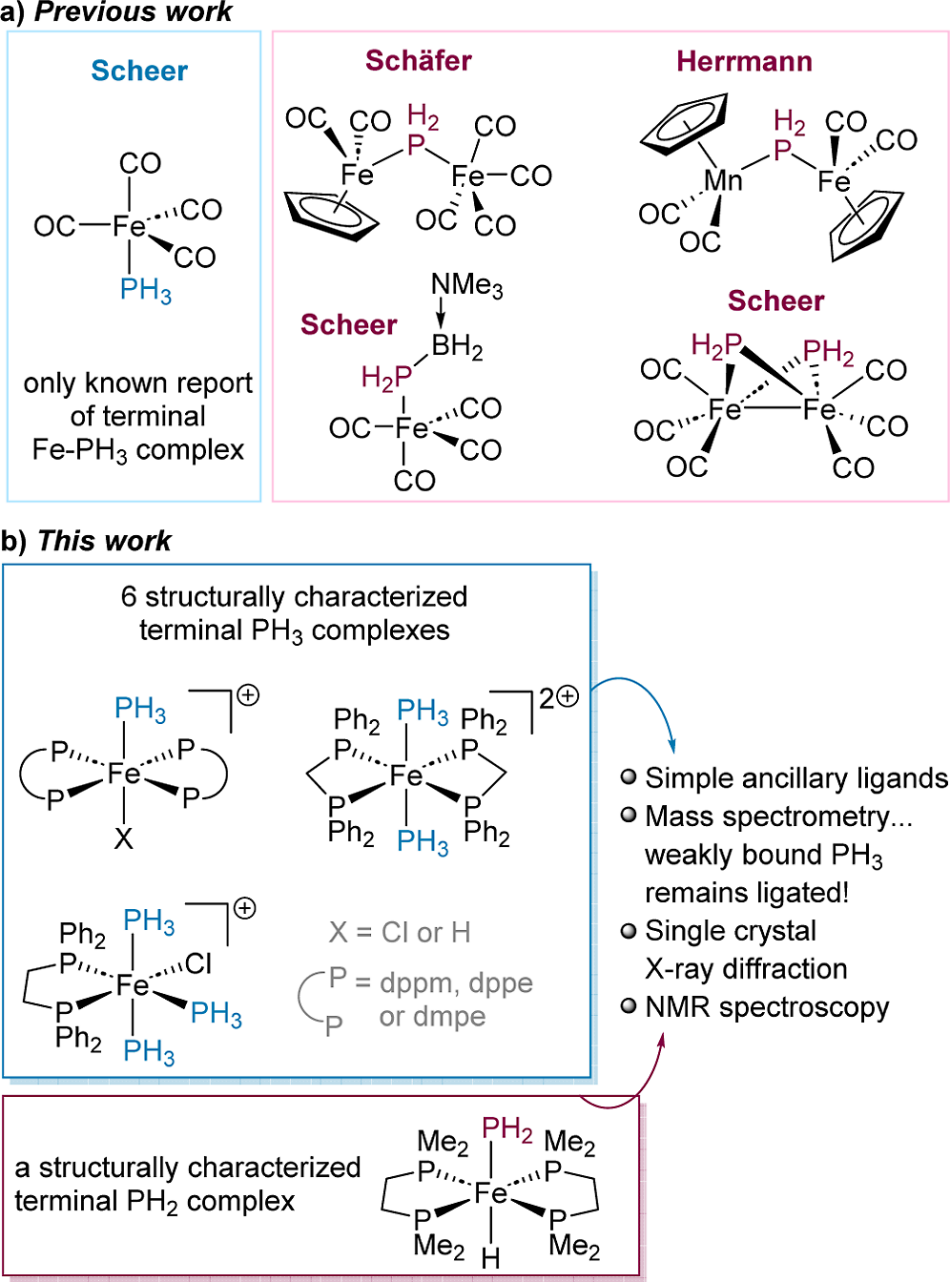
(a) Previous examples of isolated single crystal X-ray diffraction data of iron complexes bearing Fe–PH_3_ and Fe–PH_2_ motifs and (b) isolated iron(II) bisphosphine complexes from reactions with PH_3_.

The inherent dangers of working with PH_3_, a toxic and flammable gas, has meant this area of research has been restricted to those equipped with specialized equipment.^29^ However, there has been renewed interest in the use of PH_3_ as a P_1_ source with more accessible methods to form and consume PH_3_*in situ* appearing in the literature.^30−33^ Pertinent to this work, and to the best of our knowledge, there is only one report of a single crystal X-ray diffraction study of an isolated iron complex bearing the Fe–PH_3_ motif, [(CO)_4_Fe(PH_3_)] (Figure 1a).^34,35^ Similarly, although there are a handful of examples of metals (across the s-, d-, p-, and f-block) bearing terminal –PH_2_ ligands,^36−57^ there are no examples where iron is the metal; the few examples of structurally characterized iron–PH_2_ complexes exist as bridging species or employ iron as a Lewis acid to support the PH_2_ moiety.^34,35,58,59^ Notably, these iron complexes always contain CO ligands.

We herein disclose the synthesis and isolation of a library of iron bisphosphine cationic complexes bearing PH_3_ as well as the direct P–H activation of PH_3_ by [Fe(dmpe)_2_N_2_] to give a rare example of a terminal iron–phosphide complex (Figure 1b).

## Results and Discussion

Our studies on the reactivity of PH_3_ with iron bisphosphine complexes were inspired by recent work by Ball and co-workers.^31^ Their work on the application of PH_3_ demonstrated a procedurally simple method to generate and consume PH_3_*in situ* from the digestion of cheap and commercially available Zn_3_P_2_ with concentrated HCl. However, the digestion of Zn_3_P_2_ using concentrated HCl is not a suitable method for the release of PH_3_ when applications involve air- and moisture-sensitive reagents due to the presence of H_2_O, along with challenges associated with degassing. To overcome this drawback, we have modified the procedure of Ball and co-workers. Anhydrous *p*-toluenesulfonic acid (*p*-TsOH) is a suitable acid because it has a p*K*_a_ close to HCl, is a solid with a high melting point, and the ZnOTf byproduct from the digestion process is benign to the reaction conditions. Distillation from this digestion reaction in either dichloromethane or fluorobenzene shows clean generation of PH_3_ by multinuclear NMR spectroscopy (δ_P_ = −243 ppm, q, ^1^*J*_PH_ = 186 Hz) with no formation of P_2_H_4_^60^ and no detectable transfer of the acid or byproduct.

**Scheme 1:**
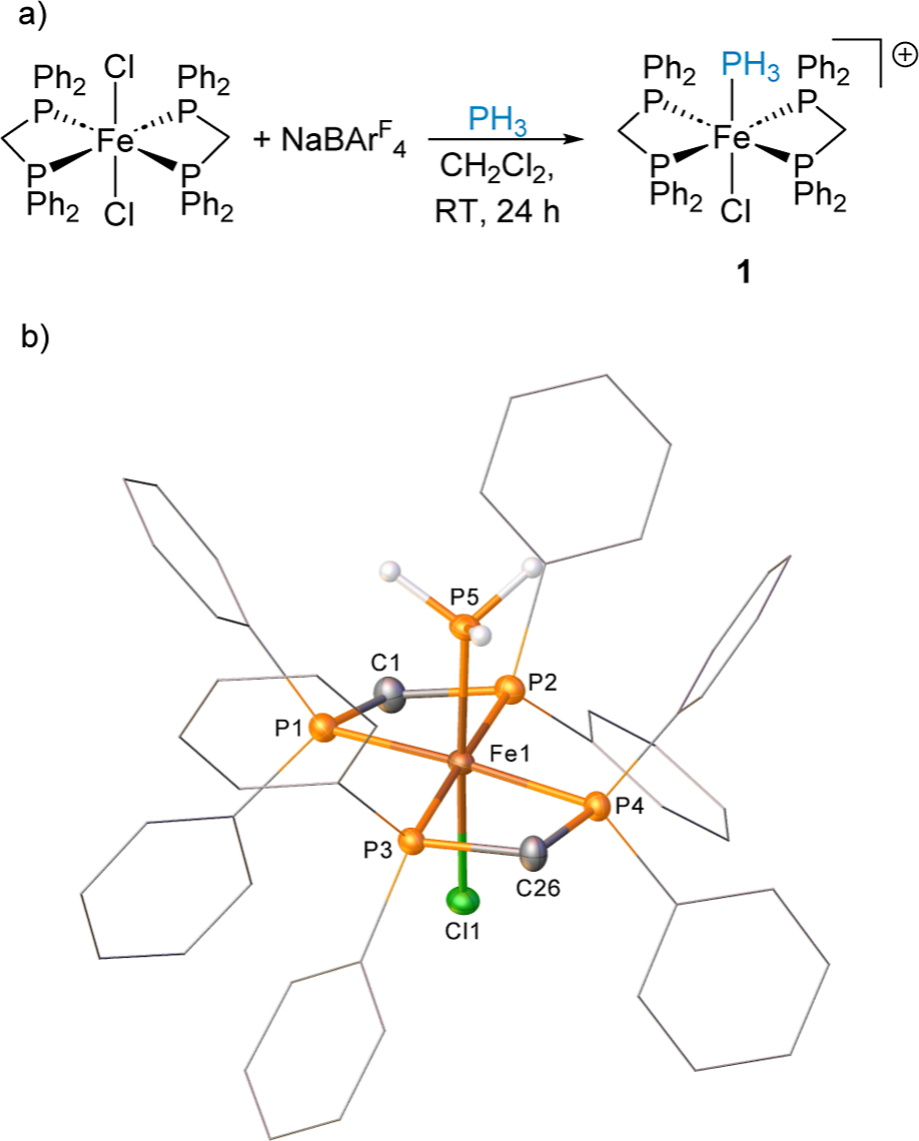
(a) Formation of **1** from Reaction of [Fe(dppm)_2_Cl_2_] with NaBAr^F^_4_ and PH_3_; The BAr^F^_4_ Counter Anion Is Removed from the Scheme for Clarity and (b) Structure of the Cation in Compound **1** (CCDC 2329812) Ellipsoids are depicted at 30% probability. Hydrogen atoms, with the exception of those, which are phosphorus-bound, have been omitted for clarity. Phenyl substituents are depicted as wireframes, also for visual ease.

Due to the poor σ-donating ability of PH_3_, we targeted cationic iron complexes with a bulky, weakly coordinating counteranion to aid isolation. One equiv of [Fe(dppm)_2_Cl_2_] reacts with 1 equiv of NaBAr^F^_4_ [Ar^F^ = 3,5-bis(trifluoromethyl)phenyl] in the presence of *in situ* generated PH_3_ in either CH_2_Cl_2_ or fluorobenzene at room temperature to generate **1** ([Fe(dppm)(Cl)(PH_3_)][BAr^F^_4_]) exclusively, which is isolated as a pink/purple crystalline solid (Scheme 1a). In CD_2_Cl_2_, an isolated sample of **1** displays two resonances in the ^31^P{^1^H} NMR spectrum, δ_P_ = 10.1 ppm (doublet, ^2^*J*_PP_ = 47.1 Hz for dppm) and δ_P_ = −64.7 ppm (pentet, ^2^*J*_PP_ = 46.1 Hz for PH_3_) which are consistent with chloride abstraction and subsequent ligation of PH_3_ to the vacant site to give the *trans*-isomer of **1**. The corresponding Fe–PH_3_ signal is also observed in the ^1^H NMR spectrum at 3.02 ppm (doublet of pentet, ^1^*J*_HP_ = 344.35 Hz and ^3^*J*_HP_ = 4.64 Hz). The signals for PH_3_ in both the ^31^P and ^1^H NMR spectra are significantly downfield compared to those of free PH_3_. In addition, ESI-MS of **1** shows a monocation without PH_3_ at *m*/*z* = 859.1421 (calcd 859.1426) with the correct isotopic pattern. This may allude to the labile nature of PH_3_ in this complex during the nebulization process. Single crystal X-ray diffraction of **1** shows an Fe1–Cl1 distance of 2.3016(7) Å and an Fe1–P5 distance of 2.1945(7) Å (Scheme 1b).

**Scheme 2:**
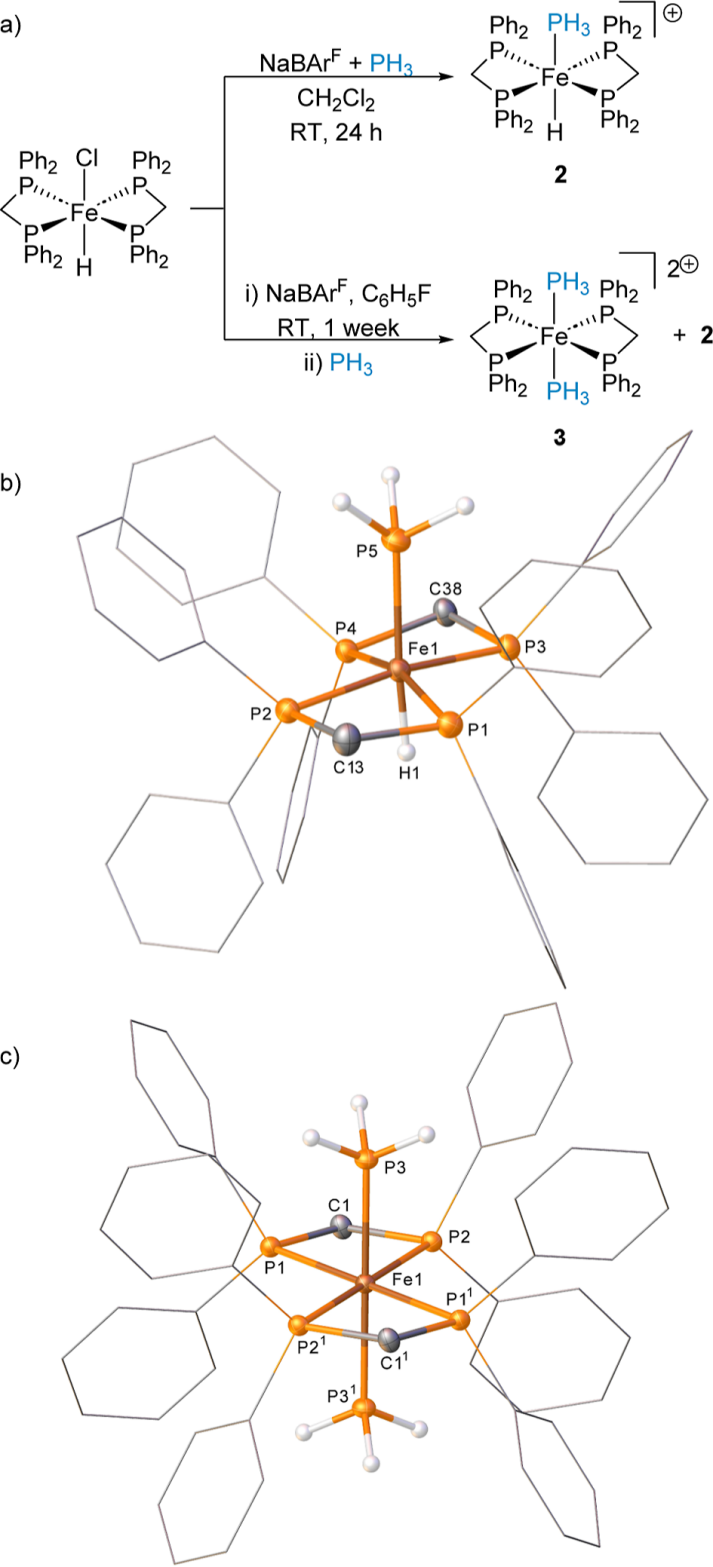
(a) Reactivity of [Fe(dppm)_2_(H)(Cl)] with NaBAr^F^_4_ and PH_3_ under CH_2_Cl_2_ and C_6_H_5_F to Give Different Product Distributions; The BAr^F^_4_ Counter Anions Are Removed from the Scheme for Clarity; (b) One of the Cations Present in the Structure of Compound **2** (CCDC 2329813)^*a*^; and (c) Structure of the Cation in Compound **3** (CCDC 2329814) Ellipsoids are depicted at 30% probability; hydrogen atoms, with the exception the hydride and of those which are phosphorus-bound, have been omitted for clarity; phenyl substituents are depicted as wireframes, also for visual ease. Ellipsoids are depicted at 30% probability. Hydrogen atoms, with the exception of those, which are phosphorus-bound, have been omitted for clarity. Phenyl substituents are depicted as wireframes, also for visual ease. Symmetry operations: 1 – *x*, 3 – *y*, 1 – *z*.

Repeating the same procedure for the monochloride analogue [Fe(dppm)_2_(H)(Cl)] gives **2** ([Fe(dppm)_2_(H)(PH_3_)][BAr^F^_4_]) exclusively when CH_2_Cl_2_ is used as the solvent (Scheme 2a). An isolated sample of complex **2** displays two resonances in the ^31^P{^1^H} NMR spectrum at δ_P_ = 30.6 ppm (doublet, ^2^*J*_PP_ = 32.7 Hz for dppm) and δ_P_ = −89.7 ppm (pentet, ^2^*J*_PP_ = 32.7 Hz for PH_3_). The retention of the iron hydride in complex **2** is observed at δ_H_ = −4.23 ppm as a pentet of doublets (^2^*J*_HP_ = 46.2 and 10.7 Hz). Here the expected *trans*-coupling, for *H*–Fe–*P*H_3_, is smaller than the *cis*-coupling for Fe–H with the four phosphorus environments associated with the two dppm ligands. This is unexpected and may allude to weak binding of the PH_3_ ligand. A direct comparison can be made with the reported iridium cationic complex, [Ir(CO)(PEt_3_)_2_(PH_2_)(PH_3_)]^+^, where the iridium hydride is assigned *trans* to the PH_3_ moiety based on coupling constants.^61^ Here Ebsworth and Mayo ascribe the ^2^*J*_HP_ = 140.7 Hz to *trans*-coupling for *H*–Ir–PH_3_ and ^2^*J*_HP_ = 9.5 Hz to *cis*-coupling for H–Ir–PH_2_. It is also worth noting there have been reports of octahedral *trans*-iron hydride phosphaalkyne complexes bearing bisphosphine ligands which show that both the *cis* and *trans*^2^*J*_HP_ of these systems can exhibit similar magnitudes.^62,63^ Pleasingly, the single crystal X-ray diffraction data for **2** allows the position of the iron hydride to be located freely on the Fourier transform map, *trans* to the PH_3_ group confirming the connectivity of **2**. Repeating the same reaction but using fluorobenzene as the solvent in the presence of PH_3_, gives a mixture of complexes **2** and **3** ([Fe(dppm)_2_(PH_3_)_2_][BAr^F^_4_]_2_), which form with varying ratios each time. Attempts to cleanly form **3** by changing the stoichiometry to 2 equiv NaBAr^F^_4_ and leaving the chloride abstraction for 1 week before addition of PH_3_ gives **3** as the major species but still with some contamination by **2**. Complex **3** is a dicationic Fe(II) complex with two PH_3_ groups ligated to the iron center *trans* to each other. Both **2** and **3** crystallize out in the same solvent system as yellow and orange crystalline materials respectively which can be mechanically separated allowing single crystal X-ray diffraction to corroborate the identity of **3** as *trans*-[Fe(dppm)_2_(PH_3_)_2_]^2+^. In contrast to complex **1**, ESI-MS of complexes **2** and **3** gives the intact monocationic and dicationic iron complexes with one and two PH_3_ still ligated to the iron centers, respectively. It is worth noting that there are even fewer reports of isolated transition metal complexes ligated by multiple PH_3_ ligands. A search of the Cambridge Structural Database reveals that five out of the seven structurally characterized complexes bear CO as ancillary ligands,^35,64−66^ the exceptions being an iridium boron cluster complex^67^ and *trans*-[RuCl_2_(PH_3_)_4_].^68^

The formation of **3** is not entirely understood under these reaction conditions but following the reaction by *in situ* NMR spectroscopy provides some insight. Addition of 1 equiv [Fe(dppm)_2_(H)(Cl)] with 1 equiv NaBAr^F^_4_ in fluorobenzene in the absence of PH_3_ results in an intractable number of signals in the ^31^P{^1^H} NMR spectrum after 1 week. A shorter reaction time does not lead to a cleaner reaction. This includes a broad signal at δ_P_ = 24 ppm. By ^1^H NMR spectroscopy a broad signal from δ_H_ = −8.50 to −9.50 ppm is observed. Both signals correspond closely to the reported signal for *trans*-[Fe(dppm)_2_H_2_] in CD_2_Cl_2_.^12^ Therefore, one can envisage that the generated 5-coordinate cationic iron intermediate, formed from the initial chloride abstraction, can react with another molecule of itself to form [Fe(dppm)_2_H_2_] and an iron dicationic intermediate that is capable of binding two PH_3_ ligands to form complex **3**. Single crystal X-ray diffraction studies on complex **2** (Scheme 2b) show little difference in the Fe1–P5 bond distance compared to **1** [2.197(1) Å in **2**, 2.1945(7) Å in **1**]. The Fe1–H1 bond distance is 1.39(3) Å. In contrast, the Fe–P3 distance in complex **3** is 2.2351(6) Å, much longer than that observed in complexes **1** and **2** (Scheme 2c).

As stated (*vide supra*), we do not observe the formation of **3** starting from [Fe(dppm)_2_Cl_2_]. This reaction exclusively forms **1** in either CH_2_Cl_2_ or C_6_H_5_F.

Selected bond angles associated with the dppm ligand for complexes **1**, **2,** and **3** are presented in Table 1. From these data, we can observe that the P–Fe–P bond angles are narrowest for complex **1**, whereas the P–C–P bond angles around the dppm ligand are wider for the *D*_2*h*_ symmetrical complex **3**.

**Table 1:** Selected Bond Angles for Complexes **1**, **2,** and **3**

complex	P1–Fe1–P2 (deg)	P3–Fe1–P4 (deg)	P1–C–P2 (deg)	P3–C–P4 (deg)
1	73.68(2)	73.85(2)	96.7(1)	95.7(1)
2	75.03(3)	74.28(3)	94.1(2)	93.2(2)
3	74.18(2)	n/a	97.2(1)	n/a

**Scheme 3:**
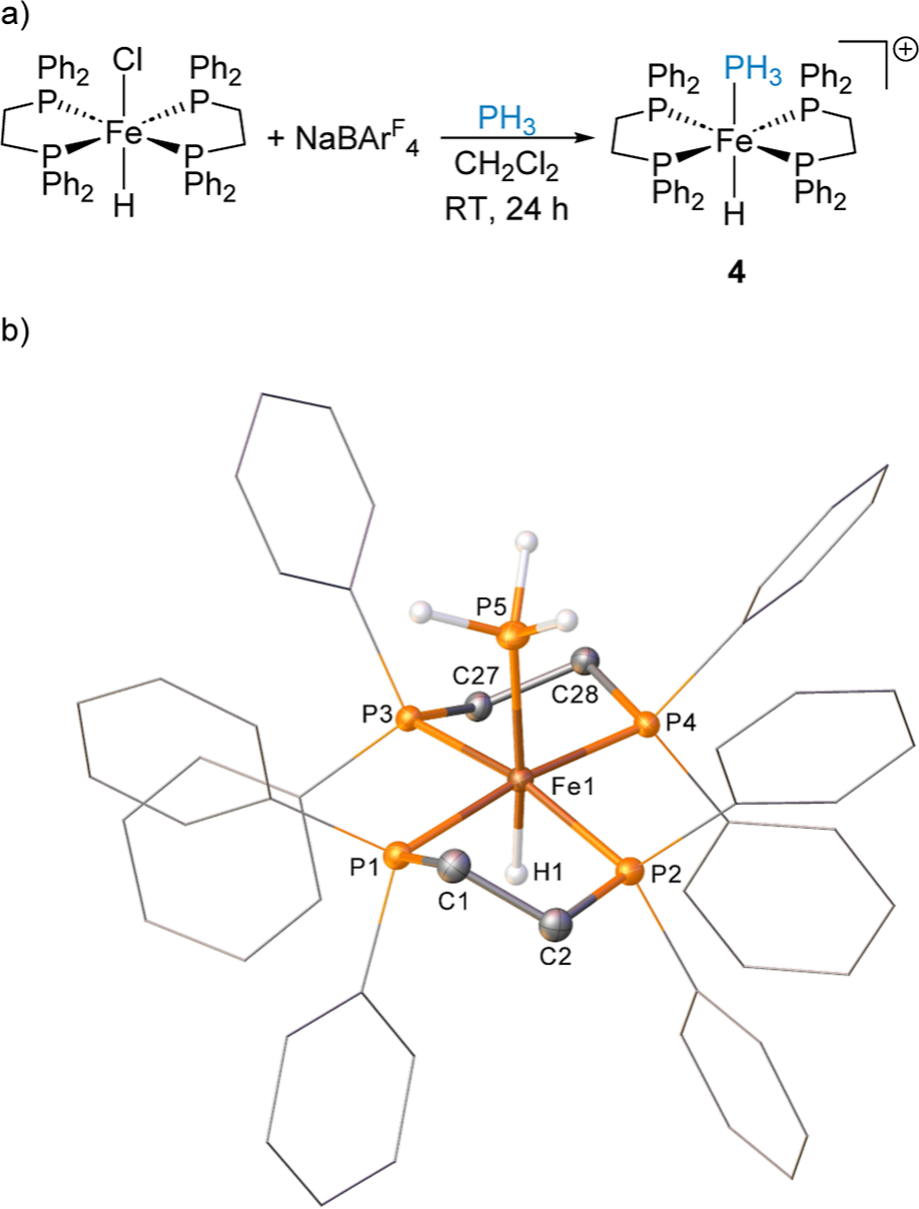
(a) Formation of **4** from Reaction of [Fe(dppe)_2_(H)(Cl)] with NaBAr^F^_4_ and PH_3_; The BAr^F^_4_ Counter Anion Is Removed from the Scheme for Clarity; and (b) Structure of the Cation in Compound **4** (CCDC 2329815) Ellipsoids are depicted at 30% probability. Hydrogen atoms, with the exception of the hydride and of those, which are phosphorus-bound, have been omitted for clarity. Phenyl substituents are depicted as wireframes, also for visual ease.

By using dppe, the reaction of 1 equiv [Fe(dppe)_2_(H)(Cl)] with 1 equiv NaBAr^F^_4_ in the presence of PH_3_ in CH_2_Cl_2_ at room temperature gives the expected cationic complex **4** ([Fe(dppe)_2_(H)(PH_3_)][BAr^F^_4_], Scheme 3a). An isolated sample of **4** displays two resonances in the ^31^P{^1^H} NMR spectrum at δ_P_ = 84.7 ppm (doublet, ^2^*J*_PP_ = 28.7 Hz for dppe) and δ_P_ = −91.4 ppm (pentet, ^2^*J*_PP_ = 28.2 Hz for PH_3_). In the ^1^H NMR spectrum, the corresponding Fe–PH_3_ signal is observed at δ_H_ = 3.24 ppm (dp, ^1^*J*_HP_ = 319.2 Hz, ^3^*J*_HP_ = 5.3 Hz), and the Fe–H signal is observed at δ_H_ = −10.99 ppm (pd, ^2^*J*_HP_ = 47.8 and 16.5 Hz). Again, the smaller *trans*-*H*–Fe–*P*H_3_ coupling is observed compared to the *cis*-coupling to dppe, akin to what is observed for complex **2**. Both single crystal X-ray diffraction and ESI-MS unambiguously confirm the assignment of complex **4** as *trans*-[Fe(dppe)_2_(H)(PH_3_)]^+^. When this reaction is repeated in fluorobenzene additional peaks are observed in both ^1^H and ^31^P{^1^H} NMR spectra showing a new species (**5**, *vide infra*) is formed in the reaction. Complex **4** can be isolated as yellow crystals with **5** forming orange crystals, allowing for mechanical separation of the two species. Single crystal X-ray diffraction studies on **4** show an elongated Fe1–H1 bond distance compared to that of the narrow bite-angle congener, **2**. Fe1–H1 in complex **4** is 1.54(2) Å, while the bond distance between Fe1 and the PH_3_ unit is also elongated at 2.2006(7) Å. Comparing the Fe1–P_dppm_ in **2** to the Fe–P_dppe_ bond distances in **4** shows slightly larger bond distances in the dppe complex, potentially indicating that dppe is more weakly coordinating and thus preventing backbonding into PH_3_, hence the larger Fe–PH_3_ bond distance. **2**: Fe1–P1 2.226(1), Fe1–P2 2.2179(9), Fe1–P3 2.2360(9), and Fe1–P4 2.2139(9) Å. **4**: Fe1–P1 2.2568(8), Fe1–P2 2.2798(6), Fe1–P3 2.2427(6), and Fe1–P4 2.2589(8) Å.

**Scheme 4:**
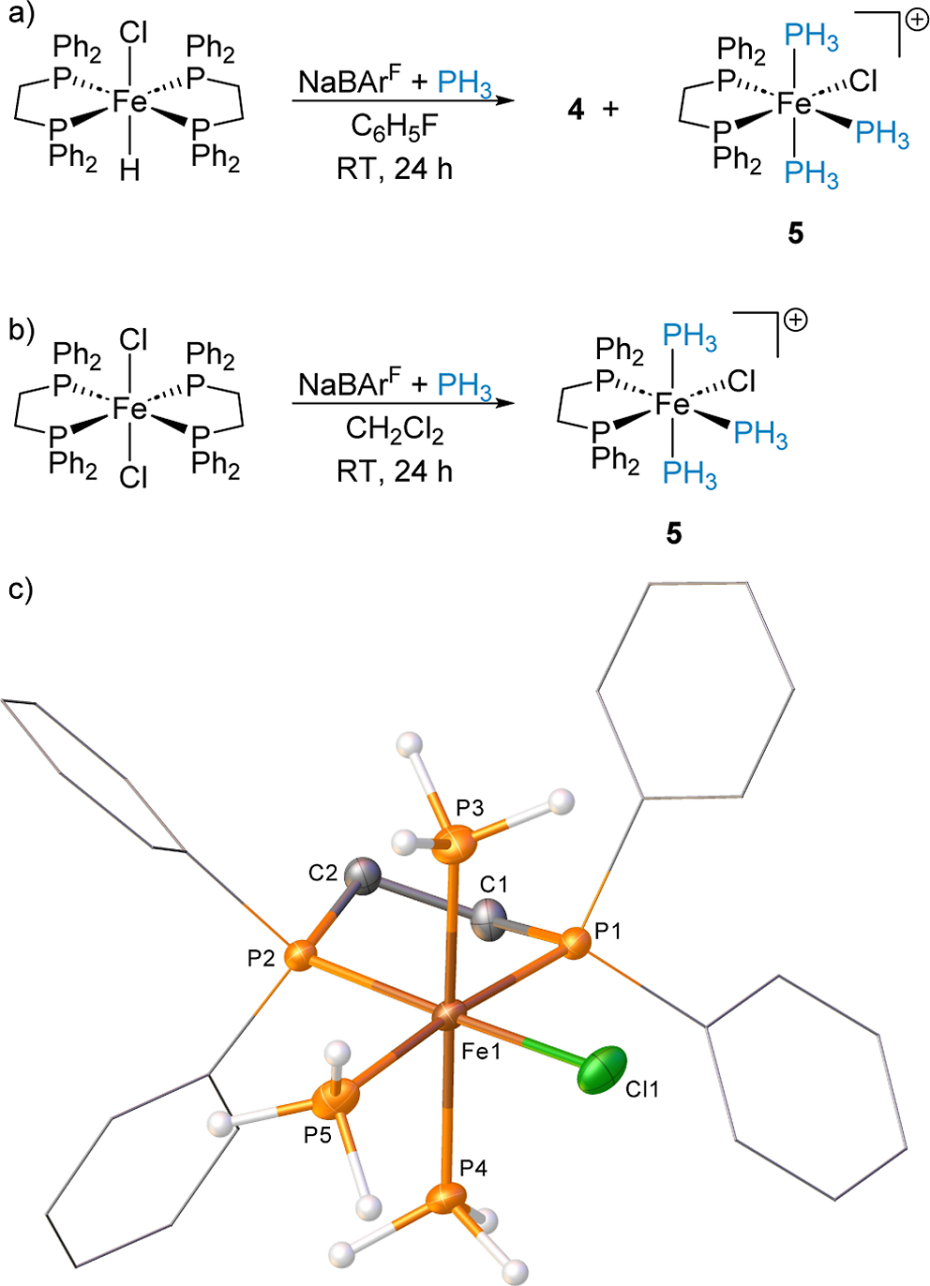
(a) Formation of **4** and **5** from [Fe(dppe)_2_(H)(Cl)]; (b) Formation of **5** from [Fe(dppe)_2_Cl_2_]; The BAr^F^_4_ Counter Anion Is Removed from the Scheme for Clarity; and (c) Structure of the Cation in Compound **5** (CCDC 2329816) Ellipsoids are depicted at 30% probability. Hydrogen atoms, with the exception of those, which are phosphorus-bound, have been omitted for clarity. Phenyl substituents are depicted as wireframes, also for visual ease.

When 1 equiv [Fe(dppe)_2_Cl_2_] is reacted with 1 equiv NaBAr^F^_4_ in either fluorobenzene or CH_2_Cl_2_ (Scheme 4a,b), the same peaks attributed to **5** ([Fe(dppe)_2_(Cl)(PH_3_)_3_][BAr^F^_4_]) are observed by multinuclear NMR spectroscopy. Characterization of this species by single crystal X-ray diffraction reveals the formation of an unusual cationic iron tris-PH_3_ complex (**5**, Scheme 4c). Complex **5** is not stable in CH_2_Cl_2_ with decomposition observed within 1 h by NMR spectroscopy through loss of PH_3_. Complex **5** displays well-resolved splitting patterns in both ^1^H and ^31^P{^1^H} NMR spectra allowing for identification of the axial and equatorial PH_3_ ligands. For example, in the ^31^P{^1^H} NMR spectrum, the equatorial-PH_3_ displays a resonance at δ_P_ = −75.4 ppm with a splitting pattern dtd; ^2^*J*_PP_ = 114.9 Hz (*trans*-PPh_2_), ^2^*J*_PP_ = 57.4 Hz (axial-PH_3_) and ^2^*J*_PP_ = 47.8 Hz (*cis*-PPh_2_). Surprisingly, ESI-MS of **5** measures a monocation at *m*/*z* = 591.0289 Da (calcd 591.0303 Da) with the correct isotopic pattern showing all three PH_3_ ligands still intact in the complex during the nebulization process. The single crystal data for this complex show that the PH_3_ units that sit *trans* to each other have longer Fe1–P distances [Fe1–P3 2.221(1) Å, Fe1–P4 2.223(1) Å], whereas the PH_3_ ligand that lies *trans* to one arm of the dppe ligand has Fe1–P5 2.263(1) Å.

**Scheme 5:**
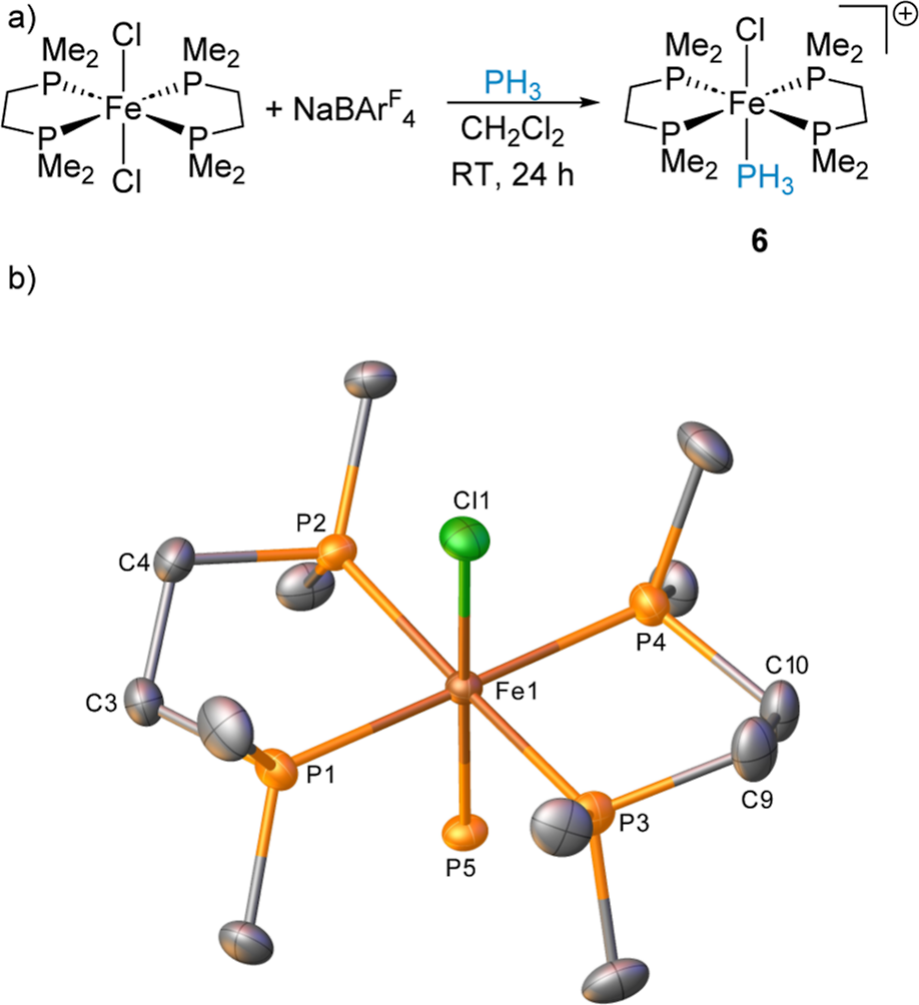
Formation of **6** from Reaction of [Fe(dmpe)_2_Cl_2_] with NaBAr^F^_4_ and PH_3_; The BAr^F^_4_ Counter Anion Is Removed from the Scheme for Clarity and (b) Structure of the Cation in Compound **6** (CCDC 2329817) Ellipsoids are depicted at 30% probability. Hydrogen atoms have been omitted for clarity. (The hydrogens attached to P5 could not be reliably located in this structure).

To investigate the effect of ancillary ligand sterics the [Fe(dmpe)_2_Cl_2_] precursor^15^ was synthesized as a direct comparison to [Fe(dppe)_2_Cl_2_]. 1 equiv [Fe(dmpe)_2_Cl_2_] reacts with 1 equiv NaBAr^F^_4_ in either CH_2_Cl_2_ or fluorobenzene resulting in the expected formation of complex **6** ([Fe(dmpe)_2_(Cl)(PH_3_)][BAr^F^_4_], Scheme 5). In CD_2_Cl_2_, the ^31^P resonance for Fe–PH_3_ is observed at δ_P_ = −64.8 ppm (pentet, ^2^*J*_PP_ = 51.9 Hz). Identification of complex **6** is further corroborated by single crystal X-ray diffraction and ESI-MS characterization. Unlike [Fe(dppe)_2_Cl_2_], which forms **5**, no additional iron species are formed in this reaction, as observed by NMR spectroscopy. A comparison of the single crystal data obtained for **6** to that obtained for the monochloride complex **1** shows that the dmpe ligand moderately influences bond metrics. For complex **1** we observe Fe1–Cl1 2.3016(7) Å, Fe1–P5 2.1945(7) Å, whereas for complex **6** we observe bond contraction at chloride with Fe1–Cl1 2.2758(7) Å and bond elongation at phosphorus with Fe1–P5 2.2127(7) Å.

**Scheme 6:**
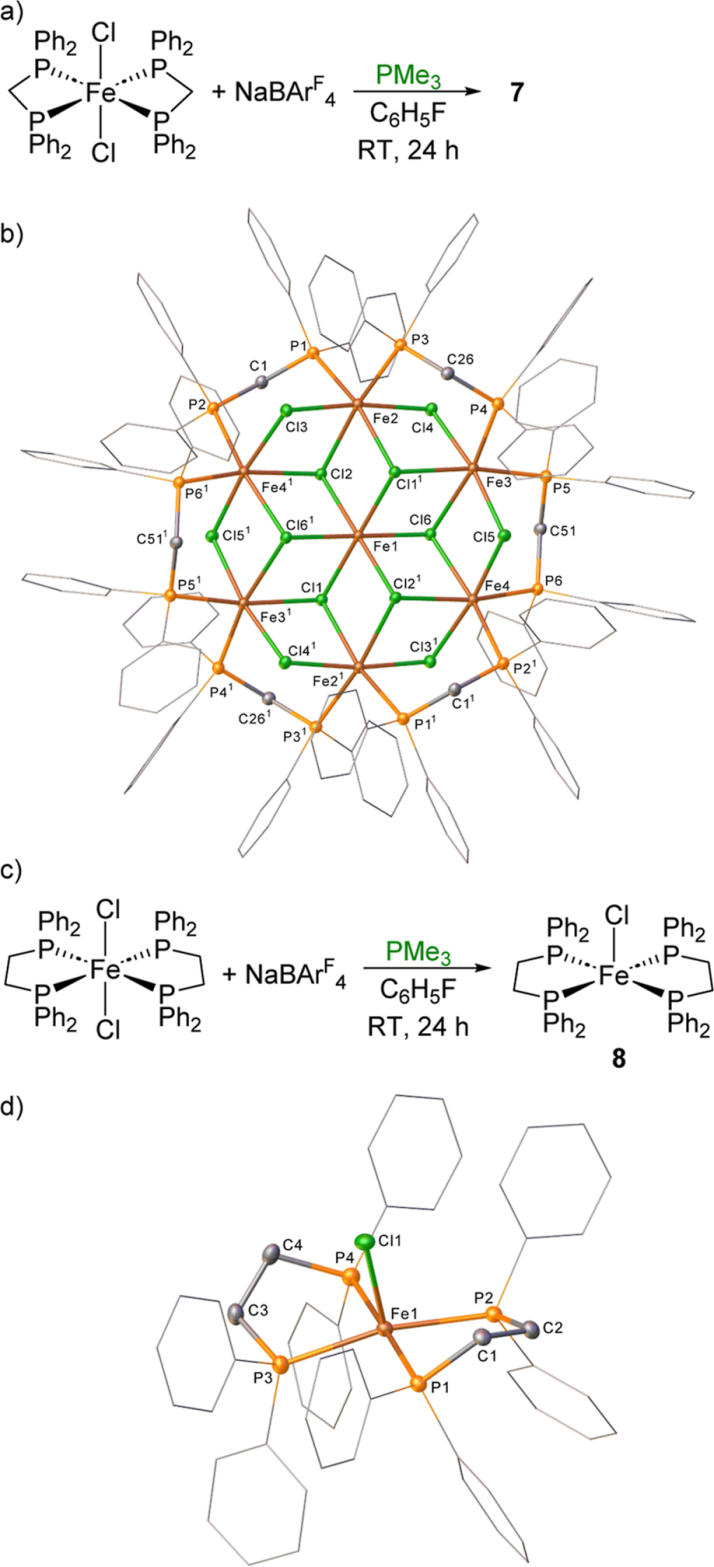
(a) Formation of Iron Macrocyclic Species **7**; (b) The Structure of the Cation in Compound **7** (CCDC 2329818); (c) Formation of Cationic 5-Coordinate Iron Complex; and (d) The Structure of the Cation in Compound **8** (CCDC 2329819) Ellipsoids Are Depicted at 30% Probability; Hydrogen Atoms Have Been Omitted for Clarity and Phenyl Substituents Are Depicted as Wireframes, Also for Visual Ease; Symmetry Operations: ^1^2 – *x*, 1 – *y*, 1 – *z*. Ellipsoids are depicted at 30% probability. Hydrogen atoms have been omitted for clarity and phenyl substituents are depicted as wireframes, also for visual ease.

The steric crowding around the iron center is further examined when [Fe(dppm)_2_Cl_2_] and [Fe(dppe)_2_Cl_2_] are reacted with NaBAr^F^_4_ and PMe_3_ (Scheme 6a,c). In both instances, no analogous 6-coordinate cationic iron species are observed, and instead, the ^1^H NMR spectra show paramagnetic signals within the spectral window +150 to −200 ppm. In the case of dppm, an unusual cationic iron cluster with one BAr^F^_4_ anion is isolated and analyzed by single-crystal X-ray diffraction analysis. Iron cluster **7** ([Fe_7_Cl_12_(dppm)_6_][BAr^F^_4_], Scheme 6b) contains seven iron centers, all in an octahedral geometry with the central iron ligated by six bridging chlorides. Alternatively, when [Fe(dppe)_2_Cl_2_] is reacted under the same conditions, the cationic intermediate 5-coordinate species **8** ([Fe(dppe)_2_(Cl)][BAr^F^_4_]) is isolated as the product from initial chloride abstraction with NaBAr^F^_4_ (Scheme 6d). Noticeable for both reactions is that the strongly σ-donating PMe_3_ ligand is not coordinated, indicating that a specific steric pocket size range favors the encapsulation of PH_3_ by these cationic iron complexes.

**Scheme 7:**
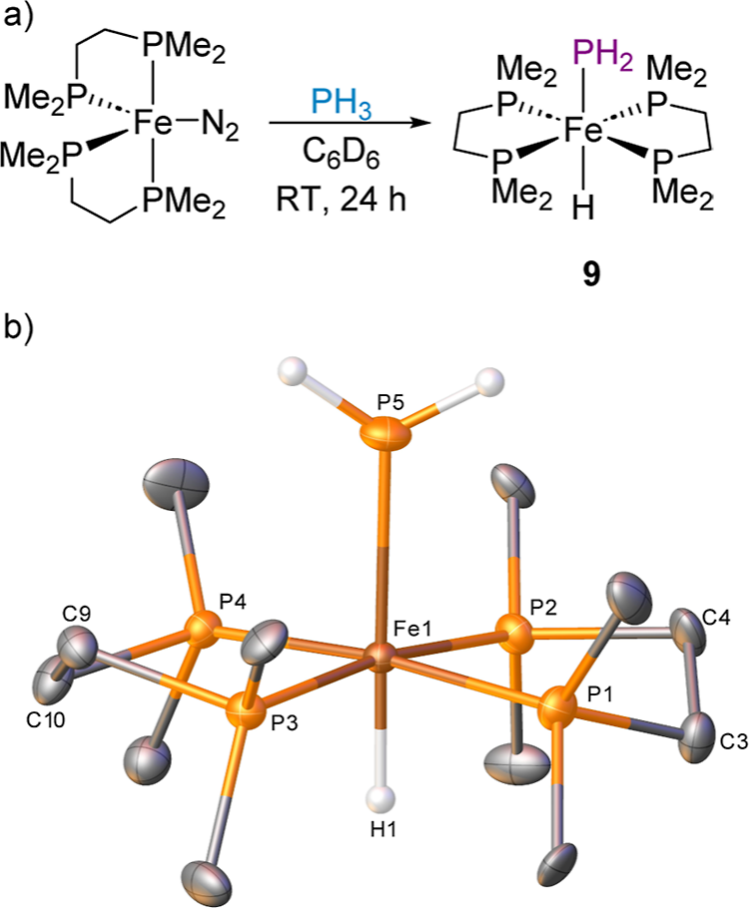
(a) P–H Activation of PH_3_ by [Fe(dmpe)_2_N_2_] Complex and (b) One of the Molecules Present in the Structure of Compound **9** (CCDC 2329820) Ellipsoids are depicted at 30% probability. Hydrogen atoms, with the exception of the hydride and of those which are phosphorus-bound, have been omitted for clarity. The disordered component has not been included, also for visual ease.

So far only cationic iron–PH_3_ complexes have been isolated; based on the high levels of reaction control and selectivity we have observed, we hypothesized that with judicious selection of the iron complex we would be able to undertake oxidative addition of PH_3_ and generate an iron–PH_2_ complex. Field and co-workers have previously reported on the reaction of [Fe(dmpe)_2_Cl_2_] with hydrazine in the presence of the reductant KC_8_ to form an intermediate Fe(0) complex.^28^ Inspired by this study, the analogous Fe(0) complex [Fe(dmpe)_2_(N_2_)] was treated with an excess of PH_3_ in C_6_D_6_. Following agitation at room temperature for 24 h, complex **9** is formed ([Fe(dmpe)_2_(H)(PH_2_)], Scheme 7). Pleasingly, rather than simple ligand substitution of N_2_ with PH_3_, activation of the P–H bond across the iron center is achieved to give a terminal iron phosphide complex. Complex **9** has been characterized by single crystal X-ray diffraction confirming the *trans*-conformation of the structure and represents the analogous structure reported by Fox and Bergman on an iron-amido complex, *trans*-[Fe(dmpe)(H)(NH_2_)].^27^ Fox and Bergman reported that both the *cis* and *trans* isomers of the iron amido complex were observed in equilibrium in a 1:4 ratio by multinuclear NMR spectroscopy. In this reaction with PH_3_, a minor peak in the ^1^H NMR spectrum of the crude reaction mixture at δ_H_ = −13.94 ppm is suspected to correspond to the Fe–H resonance for *cis*-[Fe(dmpe)_2_(H)(PH_2_)]. It is likely the *cis*-isomer forms initially in the reaction, which quickly rearranges to the more favored *trans*-isomer over time. The iron hydride is observed at δ_H_ = −18.57 ppm as a pentet (^2^*J*_HP_ = 48.14 Hz). No *trans*-PH_2_–Fe–H coupling is observed, similar to the comparative complex *trans*-[W(H)(PPh_2_)_2_(dppe)_2_] reported by Field and co-worker where only the coupling from the hydride to dppe was observed as a pentet.^69^ It has been noted *trans*-R_2_P–M–H geometries are rare and indeed only a handful have been characterized extensively.^51,69,70,71^ Complex **9** displays an Fe1–H1 distance of 1.48(4) Å and an Fe1–P5 distance of 2.337(1) Å. In comparison and as expected, Scheer’s bridged iron species displays much shorter Fe–P bond distances (Fe1–P1 2.2159 Å and Fe1–P2 2.2205 Å),^34^ which is in line with the bridging environment of the phosphide in that complex.

## Conclusions

To summarize, we have modified a procedurally simple method^31^ for the generation of PH_3_ to allow the synthesis and isolation of highly sensitive iron bisphosphine complexes. Use of NaBAr^F^_4_ to form cationic iron complexes has aided synthesis and isolation and as a result, we have isolated six Fe(II)bisphosphine–PH_3_ complexes. In one case, we have been able to prepare an iron complex bearing three PH_3_ ligands. Changing the ancillary bisphosphine ligand from dppm to dppe changes the PH_3_ coordination stoichiometry. However, changing the bisphosphine ligand has little effect on bond metrics, but when a bulkier monophosphine is employed (PMe_3_ rather than PH_3_) the effects of sterics are clear, and PMe_3_ fails to ligate. Finally, the use of an Fe(0) source has allowed us to isolate and characterize what we believe to be a very rare, or indeed unique, example of a terminal iron–PH_2_ complex. Looking forward, the PH_3_ complexes could be employed as PH_3_ transfer agents, and their reactivity compared to free-PH_3_ studied, while the Fe–PH_2_ complex could act as a catalytic intermediate in PH_2_ functionalization reactions; the onward reactivity of these complexes is currently being studied in our lab.

## Supporting Information Available

Synthetic procedures and analysis data (PDF)

## Notes

PH_3_ is a toxic and flammable gas. Use of a PH_3_ monitor is highly recommended for any synthesis involving PH_3_ in addition to the usual safety protocols in place.
