# Supplementary material for: Synthesis and Characterization of a Terminal Iron(II)–PH2 Complex and a Series of Iron(II)–PH3 Complexes
Source: Inorg Chem. 2024 Apr 2;63(15):6998–7006. doi: 10.1021/acs.inorgchem.4c00605 (PMC11022175; doi:10.1021/acs.inorgchem.4c00605)
Supplement: Supplementary file 1 — ic4c00605_si_001.pdf [file ic4c00605_si_001.pdf]

# Electronic Supporting Information

# Synthesis and Characterization of a Terminal Iron(II)-$PH_2$ Complex and a Series of Iron(II)-$PH_3$ Complexes

Samantha Lau,[a] Mary F. Mahon,[a*] and Ruth L. Webster[b*]

[a]Department of Chemistry, University of Bath, Claverton Down, Bath, BA2 7AY, UK.

[b]Yusuf Hamied Department of Chemistry, University of Cambridge, Lensfield Road, Cambridge, UK. CB2 1EW

*Email: rw740@cam.ac.uk

*Email: chsmfm@bath.ac.uk

## Table of Contents

1. General considerations ..... 2

2. Materials ..... 2

3. Generation of $PH_3$ ..... 2

3.1 Representative procedure for addition of *in situ* generated $PH_3$ ..... 2

4. Synthesis of new complexes ..... 3

4.1 Preparation of *trans*-[Fe(dppm)$_2$(Cl)($PH_3$)][BAr$^F_4$] (1) ..... 3

4.2 Preparation of *trans*-[Fe(dppm)$_2$(H)($PH_3$)][BAr$^F_4$] (2) ..... 4

4.3 Preparation of *trans*-[Fe(dppm)$_2$($PH_3$)$_2$][BAr$^F_4$]$_2$ (3) ..... 5

4.4 Preparation of *trans*-[Fe(dppe)$_2$(H)($PH_3$)][BAr$^F_4$] (4) ..... 5

4.5 Preparation of *cis*-[Fe(dppe)(Cl)($PH_3$)$_3$][BAr$^F_4$] (5) ..... 6

4.6 Preparation of *trans*-[Fe(dmpe)$_2$(Cl)($PH_3$)][BAr$^F_4$] (6) ..... 7

4.7 Preparation of *trans*-[Fe(dmpe)$_2$(H)($PH_2$)] (9) ..... 8

4.8 Synthetic procedure resulting in isolation of (7) ..... 8

4.9 Synthetic procedure resulting in isolation of (8) ..... 8

5. NMR spectra ..... 9

6. Mass Spectroscopy ..... 17

7. FT-IR spectra ..... 20

8. X-ray Crystallography data ..... 23

9. References ..... 30

## 1. General considerations

All manipulations were carried out under standard Schlenk-line and glovebox techniques under an inert atmosphere of argon (Ar) or dinitrogen ($N_2$). An MBraun MB200B glovebox was employed operating at <0.1 ppm $O_2$ and <0.1 ppm $H_2O$. Fluorobenzene ($C_6H_5F$) was pre-dried with alumina, distilled from $CaH_2$ and dried twice over 3 Å molecular sieves. $C_6D_6$ and $CD_2Cl_2$ were freeze-pump-thaw degassed × 3, dried with 3 Å molecular sieves and stored under an Ar atmosphere. Toluene was distilled from Na/benzophenone and stored over activated 3 Å molecular sieves under Ar atmosphere. Hexane, pentane and $CH_2Cl_2$ were sparged with Ar and stored over activated 3 Å molecular sieves. Glassware were dried for 12 hours at 120°C prior to use.

NMR spectra were obtained on Bruker or Agilent 400 or 500 MHz instruments, all peaks are referenced against residual solvent peak, with values quoted in ppm. NMR data were processed in Topspin or MestReNova software. UV-Vis spectra were recorded on a Mettler Toledo UV5 spectrometer. Infrared spectra were recorded at ambient temperature on a Perkin Elmer Spectrum 100 FT-IR spectrometer using a diamond ATR unit. ESI mass spectroscopy analysis were carried out on Bruker maXis-QTOF. Elemental analyses were performed at the Elemental Microanalysis laboratory, Okehampton.

## 2. Materials

Chemicals were purchased from Sigma Aldrich (Merck), Fluorochem, Acros Organics and Alfa Aesar and were used without further purification unless stated. Sodium tetrakis[3,5-bis(trifluoromethyl)phenyl]borate ($NaBAr^F{}_4$)[1] was prepared according to literature procedure. *p*-Toluenesulfonic acid monohydrate (*p*-TsOH•$H_2O$) was dried under dynamic vacuum at 120 °C over 6 h till a constant low pressure reading (~ $\times 10^{-2}$ bar) was achieved.

[Fe(dppm)$_2$Cl$_2$], [Fe(dppm)$_2$(H)(Cl)], [Fe(dppe)$_2$Cl$_2$] and [Fe(dppe)$_2$(H)(Cl)] were all prepared following the literature procedure by Aresta, Giannoccaro, Rossie and Sacco.[2] [Fe(dmpe)$_2$Cl$_2$][3] and [Fe(dmpe)$_2$N$_2$][4] were all prepared according to literature procedures.

## 3. Generation of $PH_3$

**SAFETY NOTE**: $PH_3$ gas is an extremely toxic and flammable gas. Use of a $PH_3$ monitor is highly recommended for any synthesis involving $PH_3$. Synthesis involving $PH_3$ should only be performed after appropriate training and completion of risk assessment with safety coordinator. A gas outlet to a concentrated bleach/NaOH scrubber was in operation during manipulation of this gas and all manipulations were performed in a well-ventilated fumehood or inside glovebox.

The formation of $PH_3$ was adapted from literature procedure by Ball and co-workers from the digestion of $Zn_3P_2$ with a suitable acid.[5] Based on the work by Ball and co-workers we assume digestion of $Zn_3P_2$ results in ~78% conversion to $PH_3$. Therefore, the formation of $PH_3$ was always in slight excess to the stoichiometry of iron complexes to ensure complete conversion of Fe complex.

### 3.1 Representative procedure for addition of *in situ* generated $PH_3$

In an Ar glovebox, a Teflon piston NMR tube was charged with $Zn_3P_2$ (5.2 mg, 20.1 μmol) and anhydrous *p*-toluenesulfonic acid (20.3 mg, 120 μmol) and suspended in $C_6H_5F$ or $CH_2Cl_2$ (0.5 mL) then

immediately sealed. The reaction was agitated for 16 h at room temperature in the sealed NMR tube. A second Teflon piston NMR tube was charged with [Fe] (0.03 mmol) and $NaBAr^F_4$ (26.6 mg, 0.03 mmol) and was connected *via* a short path distillation bridge to the Teflon piston NMR tube containing, the now generated, $PH_3$ (see picture below). Both Teflon piston NMR tubes were frozen at the required cryogenic temperatures (liq. $N_2$ for $CH_2Cl_2$ and toluene or dry ice/acetone for $C_6D_6$ and $C_6H_5F$) then the NMR tubes were evacuated on a high vacuum line till a stable low pressure was achieved. Under static reduced pressure, the generated $PH_3$ and the solvent were carefully distilled over to the NMR tube containing the [Fe] and $NaBAr^F_4$ cooled under low temperature. The reaction vessel, now containing all reagents, was sealed up and allowed to thaw whilst any residual $PH_3$ in the short path distillation bridge was vented through a Schlenk manifold through a bleach/NaOH scrubber.

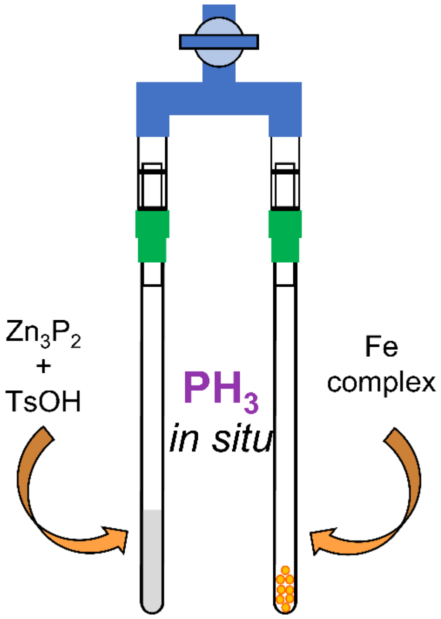


## 4. Synthesis of new complexes

### 4.1 Preparation of *trans*-[Fe(dppm)$_2$(Cl)(PH$_3$)][BAr$^F_4$] (1)

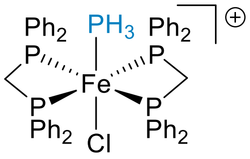

**1**

A Teflon piston NMR tube was charged with [Fe(dppm)$_2$Cl$_2$] (26.8 mg, 30.0 µmol) and $NaBAr^F_4$ (26.6 mg, 30.0 µmol). In a separate Teflon piston NMR tube, *in situ* generated $PH_3$ ($Zn_3P_2$: 5.2 mg, 20.1 µmol; *p*-TsOH: 20.6 mg, 120 µmol) and $CH_2Cl_2$ (0.5 mL) were distilled over to the solids under reduced pressure. The reaction was agitated for 24 hours at room temperature with a resulting colour change from yellow to purple red observed. The reaction was filtered through a short celite plug then layered with *n*-hexane and left to recrystallise at −30 °C to afford the title product as pink purple crystals on

diffusion, some of which were suitable for analysis using single crystal X-ray diffraction. Yield: 33.4 mg (63%).

**$^{1}$H NMR (500 MHz, $CD_2Cl_2$):** δ = 7.74–7.69 (br, 8H, ArF), 7.57–7.53 (br, 4H, ArF), 7.50 (appt t, $^{3}J_{HH}$ = 7, 6H, ArH), 7.43 (m, 2H, ArH), 7.40 (appt t, $^{3}J_{HH}$ = 8, 8H, ArH), 7.25–7.20 (br m, 20H, ArH),7.20–7.16 (br m, 4H, ArH), 5.06 (appt d vp, $^{2}J_{HH}$ = 14, $^{2}J_{HP}$ = 5, 2H, $PCH_2$), 4.99 (appt d vp, $^{2}J_{HH}$ = 13, $^{2}J_{HP}$ = 4, 2H, $PCH_2$), 3.02 (dp, $^{1}J_{HP}$ = 344.35, $^{3}J_{HP}$ = 4.64, 3H, $PH_3$). **$^{13}$C{$^{1}$H} NMR (126 MHz, $CD_2Cl_2$):** δ = 162.2 (q, $^{1}J_{CB}$ = 50, Ar$^{F}$), 135.2 (s, Ar$^{F}$), 134.1 (vt, $J_{PC}$ = 3, Ar), 132.2 (vt, $J_{PC}$ = 3, Ar), 131.7 (s, Ar), 131.2 (s, Ar), 129.7 (t, $^{1}J_{PC}$ = 3, Ar), 129.3 (q, $^{2}J_{FC}$= 31, Ar$^{F}$), 128.7 (t, $^{1}J_{PC}$ = 3, Ar), 125.0 (q, $^{1}J_{FC}$= 273, $CF_3$), 117.9 (sept, $^{3}J_{FC}$ = 4, Ar$^{F}$), 45.5 (br m, $CH_2$). **$^{31}$P{$^{1}$H} NMR (202 MHz, $CD_2Cl_2$):** δ = 10.1 (d, $^{2}J_{PP}$ = 47.1, dppm), −64.7 (p, $^{2}J_{PP}$ = 46.1, $PH_3$). **$^{19}$F{$^{1}$H} NMR (470 MHz, $CD_2Cl_2$)**: δ −62.9 (s, $CF_3$). **Elemental Analysis** calc. for $C_{82}H_{59}BClF_{24}FeP_5$: C, 56.05; H, 3.38; N, 0.00. Found: C, 55.13; H, 3.39; N, <0.05. **HR ESI-MS** (250 °C, 5.5 kV, positive ion): 859.1421, [*M*−$PH_3$]$^{+}$ (calc. 859.1426) *m/z*.

## 4.2 Preparation of *trans*-[Fe(dppm)$_2$(H)($PH_3$)][BAr$^{F}_4$] (2)

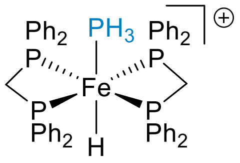


**2**

A Teflon piston NMR tube was charged with [Fe(dppm)$_2$(H)(Cl)] (25.8 mg, 30.0 µmol) and NaBAr$^{F}_4$ (26.6 mg, 30.0 µmol). In a separate Teflon piston NMR tube, *in situ* generated $PH_3$ ($Zn_3P_2$: 5.2 mg, 20.1 µmol; *p*-TsOH: 20.6 mg, 120 µmol) and $CH_2Cl_2$ (0.5 mL) were distilled over to the solids under reduced pressure. The reaction was agitated for 24 hours at room temperature with a resulting colour change from mauve to yellow observed. The reaction was filtered through a short celite plug then layered with *n*-hexane and left to recrystallise at −30 °C to afford the title product as yellow crystals on diffusion, some of which were suitable for analysis using single crystal X-ray diffraction. Yield: 22.9 mg (44%).

**$^{1}$H NMR (500 MHz, $CD_2Cl_2$):** δ = 7.74–7.69 (br, 8H, ArF), 7.57–7.54 (br, 4H, ArF), 7.46 (appt t, $^{3}J_{HH}$ = 8, 4H, ArH), 7.39 (appt t, $^{3}J_{HH}$ = 7, 4H, ArH), 7.33 (appt t, $^{3}J_{HH}$ = 8, 8H, ArH), 7.31–7.25 (br, 8H, ArH), 7.19 (appt t, $^{3}J_{HH}$ = 8, 8H, ArH), 6.95 (br appt t, $^{3}J_{HH}$ = 8, 8H, ArH), 4.68–4.58 (m, 2H, $PCH_2$), 4.34 (appt dt, $^{2}J_{HH}$ = 11, $^{2}J_{HP}$ = 5.66, 2H, $PCH_2$), 3.90 (dp, $^{1}J_{HP}$ = 321.93, $^{3}J_{HP}$ = 6.04, 3H, $PH_3$), −4.23 (pd, $^{2}J_{HP}$ = 46.24 and 10.68, 1H, Fe–H). **$^{13}$C{$^{1}$H} NMR (126 MHz, $CD_2Cl_2$):** δ = 162.2 (q, $^{1}J_{CB}$ = 50, Ar$^{F}$), 135.2 (s, Ar$^{F}$), 132.6 (vt, $J_{PC}$ = 6, Ar), 131.7 (vt, $J_{PC}$ = 6, Ar), 131.0 × 2 (s, Ar), 129.4 (t, $^{1}J_{PC}$ = 4, Ar), 129.3 (q, $^{2}J_{FC}$= 31, Ar$^{F}$), 129.1 (t, $^{1}J_{PC}$ = 5, Ar), 125.0 (q, $^{1}J_{FC}$= 273, $CF_3$), 117.9 (sept, $^{3}J_{FC}$ = 4, Ar$^{F}$), 51.2 × 2 (br m, $CH_2$). **$^{31}$P{$^{1}$H} NMR (202 MHz, $CD_2Cl_2$):** δ = 30.6 (d, $^{2}J_{PP}$ = 32.7, dppm), −89.7 (p, $^{2}J_{PP}$ = 32.7, $PH_3$). **$^{19}$F{$^{1}$H} NMR (470 MHz, $CD_2Cl_2$)**: δ −62.9 (s, $CF_3$). **Elemental Analysis** calc. for $C_{82}H_{60}BF_{24}FeP_5(CH_2Cl_2)$: C, 55.10; H, 3.29; N, 0.00. Found: C, 55.21; H, 3.19; N, <0.05. **HR ESI-MS** (250 °C, 5.5 kV, positive ion): 859.1859, [*M*]$^{+}$ (calc. 859.1788) *m/z*.

## 4.3 Preparation of *trans*-[Fe(dppm)$_2$(PH$_3$)$_2$][BAr$^F{}_4$]$_2$ (3)

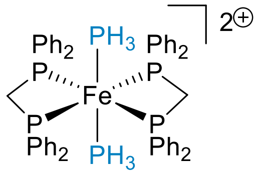

**3**

A Teflon piston NMR tube was charged with [Fe(dppm)$_2$(H)(Cl)] (12.9 mg, 15.0 µmol) and NaBAr$^F{}_4$ (26.6 mg, 30.0 µmol) and dissolved in $C_6H_5F$ (0.3 mL). The reaction was left to agitate at room temperature for 1 week resulting in a colour change from mauve to dark orange with a lot of precipitate. In a separate Teflon piston NMR tube, *in situ* generated $PH_3$ ($Zn_3P_2$: 5.2 mg, 20.1 µmol; *p*-TsOH: 20.6 mg, 120 µmol) and fluorobenzene (0.5 mL) were distilled over to the [Fe] under reduced pressure. The reaction was agitated for 24 hours at room temperature with a resulting colour change from dark orange to orangey-yellow observed. The reaction was filtered through a short celite plug then layered with *n*-hexane and left to recrystallise at −30 °C to afford the title product as orange crystals on diffusion with some contamination of *trans*-[Fe(dppm)$_2$(H)(PH$_3$)][BAr$^F{}_4$] as yellow crystals. Mechanical separation of these two products was achieved to allow for single crystal X-ray diffraction of the title compound.

**$^1$H NMR (400 MHz, CD$_2$Cl$_2$):** δ = 7.75–7.68 (br, 16H, ArF), 7.61 (appt t, $^3J_{HH}$ = 8, 4H, ArH), 7.56–7.50 (br, 8H, ArF), 7.46 (appt t, $^3J_{HH}$ = 8, 8H, ArH), 7.41–7.27 (m, 12H, ArH), 7.19 (appt t, $^3J_{HH}$ = 7, 2H, ArH), 7.15 (appt t, $^3J_{HH}$ = 7, 2H, ArH), 7.10–7.01 (m, 8H, ArH), 6.98–6.91 (br, 4H, ArH), 4.90 (appt t, $^2J_{HP}$ = 9, 4H, PCH$_2$), 4.18 (appt dp, $^1J_{HP}$ = 365, $^3J_{HP}$ = 8.37, 6H, PH$_3$). **$^{31}$P{$^1$H} NMR (162 MHz, CD$_2$Cl$_2$):** δ = 14.0 (t, $^2J_{PP}$ = 54.5, dppm), −65.2 (p, $^2J_{PP}$ = 54.4, PH$_3$). **$^{19}$F{$^1$H} NMR (471 MHz, CD$_2$Cl$_2$)**: δ −63.0 (s, CF$_3$). **HR ESI-MS** (250 °C, 5.5 kV, positive ion): 446.0833, [*M*]$^{2+}$ (calc. 446.0838) *m/z*.

Unfortunately, unsatisfactory elemental analysis was obtained for complex **3**.

## 4.4 Preparation of *trans*-[Fe(dppe)$_2$(H)(PH$_3$)][BAr$^F{}_4$] (4)

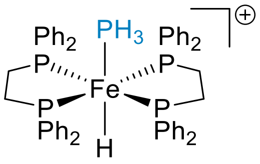

**4**

A Teflon piston NMR tube was charged with [Fe(dppe)$_2$(H)(Cl)] (13.3 mg, 15.0 µmol) and NaBAr$^F{}_4$ (13.3 mg, 15.0 µmol). In a separate Teflon piston NMR tube, *in situ* generated $PH_3$ ($Zn_3P_2$: 2.6 mg, 10.4 µmol; *p*-TsOH: 10.3 mg, 59.8 µmol) and $CH_2Cl_2$ (0.5 mL) were distilled over to the solids under reduced pressure. The reaction was agitated for 24 hours at room temperature with a resulting colour change from pink to yellowy orange observed. The reaction was filtered through a short celite plug then layered with *n*-hexane and left to recrystallise at −30 °C to afford the title product as yellow crystals on diffusion, some of which were suitable for analysis using single crystal X-ray diffraction. Yield: 14.0 mg (53%).

**$^1$H NMR (500 MHz, $CD_2Cl_2$):** δ = 7.74–7.69 (br, 8H, ArF), 7.66–7.59 (br, 8H, ArH), 7.57–7.53 (br, 4H, ArF), 7.39 (appt t, $^3J_{HH}$ = 7, 4H, ArH), 7.32 (appt t, $^3J_{HH}$ = 7, 4H, ArH), 7.21 (appt t, $^3J_{HH}$ = 8, 8H, ArH), 7.17 (appt t, $^3J_{HH}$ = 8, 8H, ArH), 6.51–6.44 (br, 8H, ArH), 3.24 (dp, $^1J_{HP}$ = 319.24, $^3J_{HP}$ = 5.32, 3H, $PH_3$), 2.52–2.34 (br m, 4H, $PCH_2$), 2.19–2.00 (br m, 4H, $PCH_2$) , −10.99 (pd, $^2J_{HP}$ = 47.83 and 16.54, 1H, Fe–H). **$^{13}$C{$^1$H} NMR (126 MHz, $CD_2Cl_2$):** δ = 162.2 (q, $^1J_{CB}$ = 50, $Ar^F$), 135.2 (s, $Ar^F$), 133.5 (s, Ar), 132.5 (s, Ar), 130.9 (s, Ar), 130.7 (s, Ar), 129.3 (q, $^2J_{FC}$= 31, $Ar^F$), 129.2 (vt, $^1J_{PC}$ = 2, Ar), 128.3 (vt, $^1J_{PC}$ = 2, Ar), 125.0 (q, $^1J_{FC}$= 273, $CF_3$), 117.9 (sept, $^3J_{FC}$ = 4, $Ar^F$) 34.1 (m, $CH_2$) 34.0 (m, $CH_2$). **$^{31}$P{$^1$H} NMR (202 MHz, $CD_2Cl_2$):** δ = 84.7 (d, $^2J_{PP}$ = 28.7, dppe), −91.4 (p, $^2J_{PP}$ = 28.2, $PH_3$). **$^{19}$F{$^1$H} NMR (470 MHz, $CD_2Cl_2$)**: δ −62.9 (s, $CF_3$). **Elemental Analysis** calc. for $C_{84}H_{64}BF_{24}FeP_5(CH_2Cl_2)$: C, 55.61; H, 3.62; N, 0.00. Found: C, 55.37; H, 3.32; N, <0.05. **HR ESI-MS** (250 °C, 5.0 kV, positive ion): 887.2193, $[M]^+$ (calc. 887.2101) *m/z*.

## 4.5 Preparation of *cis*-[Fe(dppe)(Cl)($PH_3$)$_3$][BAr$^F_4$] (5)

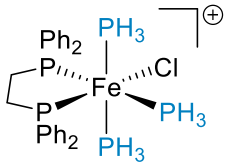


**5**

**Method A**: A Teflon piston NMR tube was charged with [Fe(dppe)$_2$(H)(Cl)] (13.3 mg, 15.0 μmol) and NaBAr$^F_4$ (13.3 mg, 15.0 μmol). In a separate Teflon piston NMR tube, *in situ* generated $PH_3$ ($Zn_3P_2$: 2.6 mg, 10.4 μmol; *p*-TsOH: 10.3 mg, 59.8 μmol) and $C_6H_5F$ (0.5 mL) were distilled over to the solids under reduced pressure. The reaction was agitated for 24 hours at room temperature with a resulting colour change from pink to yellowy orange observed. The reaction was filtered through a short celite plug then layered with *n*-hexane and left to recrystallise at −30 °C over a protracted time frame to afford trans-[Fe(dppe)$_2$(H)($PH_3$)][BAr$^F_4$] as yellow crystals and the title compound as orange crystals. Mechanical separation of these two products was achieved to allow for single crystal X-ray diffraction of the title compound. Yield: 4.0 mg (18%).

**Method B**: A Teflon piston NMR tube was charged with [Fe(dppe)$_2$Cl$_2$] (13.9 mg, 15.0 μmol) and NaBAr$^F_4$ (13.3 mg, 15.0 μmol). In a separate Teflon piston NMR tube, *in situ* generated $PH_3$ ($Zn_3P_2$: 2.6 mg, 10.4 μmol; *p*-TsOH: 10.3 mg, 59.8 μmol) and $CH_2Cl_2$ (0.5 mL) were distilled over to the solids under reduced pressure. The reaction was agitated for 24 hours at room temperature with a resulting colour change from pink to orange observed. The reaction was filtered through a short celite plug then layered with *n*-hexane and left to recrystallise at −30 °C over a protracted time frame to afford the title compound as orange crystals on diffusion, some of which were suitable for analysis using single crystal X-ray diffraction. Yield: 6.2 mg (28%).

**$^1$H NMR (500 MHz, $CD_2Cl_2$):** δ = 7.74–7.70 (br, 8H, ArF), 7.58–7.54 (br, 4H, ArH), 7.51–7.41 (br, 20H, Ar), 7.08 (appt t, $^3J_{HH}$ = 7, 4H, $PCH_2$), 4.50 (dt, $^1J_{HP}$ = 341.08, $^3J_{HP}$ = 11.78, 3H, equatorial-$PH_3$), 3.49 (dddd, $^1J_{HP}$ = 350.93, $^3J_{HP}$ = 12.34, $^3J_{HP}$ = 7.85, $^3J_{HP}$ = 3.37, 6H, apical-$PH_3$). **$^{31}$P{$^1$H} NMR (202 MHz,**

**$CD_2Cl_2$):** δ = 89.7 (appt qd, $^{2}J_{PP}$ = 49, $^{3}J_{PP}$ = 20.3, dppe), 63.5 (dtd, $^{2}J_{PP}$ =112.1, $^{2}J_{PP}$ = 64.4, $^{3}J_{PP}$ = 20.3, dppe), −65.9 (appt q, $^{2}J_{PP}$ = 62, apical-$PH_3$), −75.4 (dtd, $^{2}J_{PP}$ =114.9, $^{2}J_{PP}$ = 57.4, $^{2}J_{PP}$ = 47.8, equatorial-$PH_3$). **$^{19}F\{^1H\}$ NMR (470 MHz, $CD_2Cl_2$)**: δ −62.9 (s, $CF_3$). **HR ESI-MS** (250 °C, 5.5 kV, positive ion): 591.0289, [*M*]$^+$ (calc. 591.0303) *m/z*.

Unable to obtain a satisfactory $^{13}C\{^1H\}$ NMR spectrum of *cis*-[Fe(dppe)(Cl)$(PH_3)_3$][$BAr^F_4$] due to instability in $CD_2Cl_2$ overtime with decomposition to unknown compound over 1 hour and loss of $PH_3$ seen by $^{31}P\{^1H\}$ NMR spectroscopy. Unfortunately, unsatisfactory elemental analysis was obtained for complex **5**.

## 4.6 Preparation of *trans*-[Fe(dmpe)$_2$(Cl)($PH_3$)][$BAr^F_4$] (6)

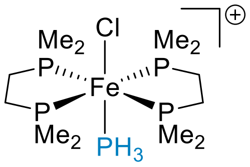


**6**

A Teflon piston NMR tube was charged with [Fe(dmpe)$_2Cl_2$] (12.8 mg, 30.0 μmol) and $NaBAr^F_4$ (26.6 mg, 30.0 μmol). In a separate Teflon piston NMR tube, *in situ* generated $PH_3$ ($Zn_3P_2$: 5.2 mg, 20.1 μmol; *p*-TsOH: 20.6 mg, 120 μmol) and $C_6H_5F$ (0.5 mL) were distilled over to the solids under reduced pressure. The reaction was agitated for 24 hours at room temperature with a resulting colour change from turquoise green to orange pink observed. The reaction was filtered through a short celite plug then layered with *n*-hexane and left to recrystallise at −30 °C to afford the title product as orangey pink crystals on diffusion, some of which were suitable for analysis using single crystal X-ray diffraction. Yield: 33.9 mg (88%).

**$^1H$ NMR (500 MHz, $CD_2Cl_2$):** δ = 7.74–7.69 (br, 8H, ArF), 7.58–7.53 (br, 4H, ArF), 3.03 (dp, $^{1}J_{HP}$ = 323.73, $^{3}J_{HP}$ = 5.67, 3H, $PH_3$), 2.11 (vp appt d, $^{2}J_{HP}$ = 9, $^{2}J_{HH}$ = 4, 4H, $PCH_2$), 1.83 (vp appt d, $^{2}J_{HP}$ = 8, $^{2}J_{HH}$ = 4, 4H, $PCH_2$), 1.55 (vp, $J_{HP}$ = 2, 12H, $P(CH_3)_2$), 1.45 (br s, 12H, $P(CH_3)_2$). **$^{13}C\{^1H\}$ NMR (126 MHz, $CD_2Cl_2$):** δ = 162.2 (q, $^{1}J_{CB}$ = 50, $Ar^F$), 135.2 (s, $Ar^F$), 129.3 (q, $^{2}J_{FC}$= 31, $Ar^F$), 125.0 (q, $^{1}J_{FC}$= 273, $CF_3$), 117.9 (br s, $Ar^F$), 29.7 (p, $^{1}J_{PC}$ = 12.9, $CH_2$), 14.5 (p, $^{1}J_{PC}$ = 6.7, $CH_3$), 13.9 (p, $^{1}J_{PC}$ = 6.6, $CH_3$). **$^{31}P\{^1H\}$ NMR (202 MHz, $CD_2Cl_2$):** δ = 56.1 (d, $^{2}J_{PP}$ = 52.1, dmpe), −74.8 (p, $^{2}J_{PP}$ = 51.9, $PH_3$). **$^{19}F\{^1H\}$ NMR (470 MHz, $CD_2Cl_2$)**: δ −62.8 (s, $CF_3$). **Elemental Analysis** calc. for $C_{44}H_{47}BClF_{24}FeP_5$: C, 41.01; H, 3.68; N, 0.00. Found: C, 41.39; H, 3.42; N, <0.05. **HR ESI-MS** (250 °C, 5.5 kV, positive ion): 425.0500, [*M*]$^+$ (calc. 425.0459) *m/z*.

## 4.7 Preparation of *trans*-[Fe(dmpe)$_2$(H)(PH$_2$)] (9)

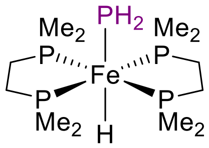

**9**

A Teflon piston NMR tube was charged with [Fe(dmpe)$_2$N$_2$] (11.2 mg, 29.2 μmol). In a separate Teflon piston NMR tube, *in situ* generated PH$_3$ (Zn$_3$P$_2$: 5.2 mg, 20.1 μmol; *p*-TsOH: 20.6 mg, 120 μmol) and toluene (0.5 mL) were distilled over to [Fe(dmpe)$_2$N$_2$] under reduced pressure. The reaction was agitated for 24 hours at room temperature with a resulting colour change from red orange to light orange observed. The reaction was filtered through a short celite plug then layered with *n*-hexane and left to recrystallise at −30 °C to afford the title product as orange crystals on diffusion, some of which were suitable for analysis using single crystal X-ray diffraction. Yield: 11.4 mg (76%).

**$^1$H NMR (400 MHz, C$_6$D$_6$):** δ = 1.69 (br m, 4H, PCH$_2$), 1.38 (s, 12H, PCH$_3$), 1.24 (br m, 4H, PCH$_2$), 1.14 (s, 12H, PCH$_3$), −0.41 (dp, $^1J_{HP}$ = 154.42, $^3J_{HP}$ = 8.57, 2H, PH$_2$), −18.57 (p, $^2J_{HP}$ = 48.14, 1H, Fe–H). **$^{13}$C{$^1$H} NMR (126 MHz, C$_6$D$_6$):** δ = 32.1 (vp, $J_{PC}$ = 14, CH$_2$), 26.5 (br m, CH$_3$), 16.0 (br m, CH$_3$). **$^{31}$P{$^1$H} NMR (202 MHz, C$_6$D$_6$):** δ = 73.1 (d, $^2J_{PP}$ = 8.7, dmpe), −172.6 (br p, $^2J_{PP}$ = 7.2, PH$_2$). **$^{31}$P NMR (162 MHz, C$_6$D$_6$):** δ = 73.1 (br d, $^2J_{HP}$ = 48.4, dmpe), −172.5 (t, $^1J_{HP}$ = 157.0, PH$_2$). **HR ESI-MS** (250 °C, 5.0 kV, positive ion): 391.0859, [*M*+*H*]$^+$ (calc. 391.0849) *m/z*.

Unfortunately, unsatisfactory elemental analysis was obtained for complex **9**.

## 4.8 Synthetic procedure resulting in isolation of (7)

A Teflon piston NMR tube was charged with [Fe(dppm)$_2$Cl$_2$] (13.4 mg, 15.0 μmol), NaBAr$^F_4$ (13.3 mg, 15.0 μmol) and C$_6$H$_5$F (0.5 mL) resulting in a milkshake yellow solution. PMe$_3$ (1.5 μL, 15.0 μmol) was added to the reaction giving a light yellow solution with a lot of white precipitate. The reaction was agitated for 24 hours at room temperature. $^1$H NMR spectrum shows paramagnetic signals within the spectral window +150 to −200 ppm. The reaction was filtered through a short celite plug and layered with *n*-hexane and left to recrystallise at −30 °C over a protracted time frame to afford **7** as colourless crystals.

## 4.9 Synthetic procedure resulting in isolation of (8)

A Teflon piston NMR tube was charged with [Fe(dppe)$_2$Cl$_2$] (13.9 mg, 15.0 μmol), NaBAr$^F_4$ (13.3 mg, 15.0 μmol) and C$_6$H$_5$F (0.5 mL) resulting in a light orange solution. PMe$_3$ (1.5 μL, 15.0 μmol) was added to the reaction forming a darker orange solution. The reaction was agitated for 24 hours at room temperature. $^1$H NMR spectrum shows paramagnetic signals within the spectral window +150 to −200 ppm. Solvent removed *in vacuo* and the reaction was redissolved in CH$_2$Cl$_2$ then filtered through a short celite plug and layered with *n*-hexane and left to recrystallise at −30 °C over a protracted time frame to afford **8** as orange crystals.

## 5. NMR spectra

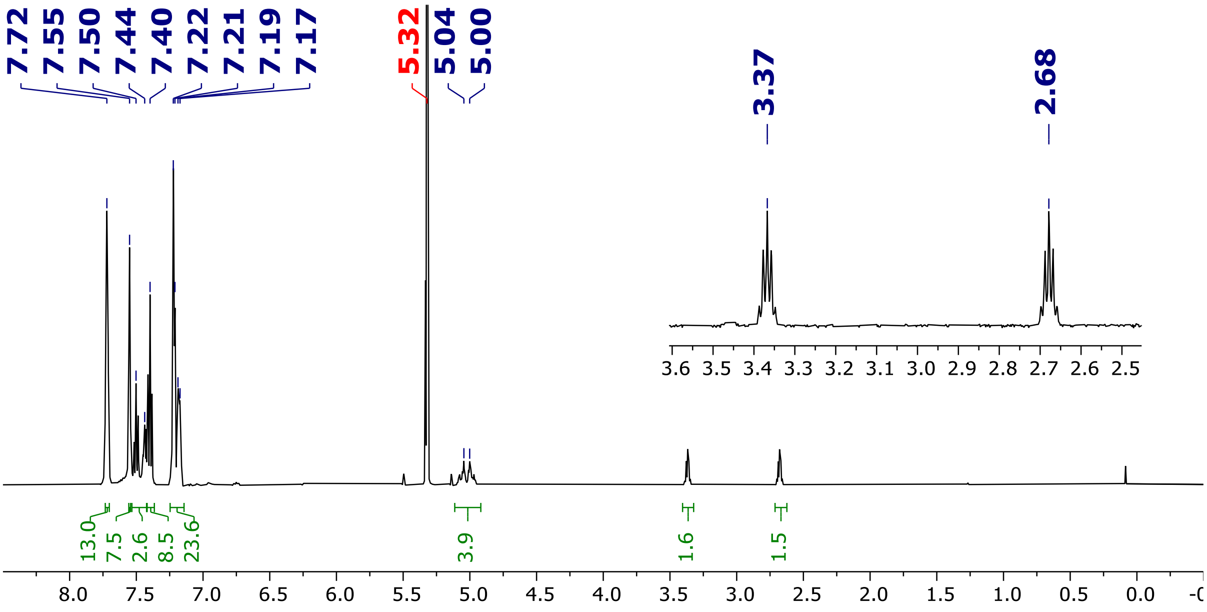


**Figure S 1.** $^{1}H$ NMR spectrum (500 MHz, 298 K, $CD_2Cl_2$) *trans*-[Fe(dppm)$_2$(Cl)($PH_3$)][BAr$^F_4$] (**1**)

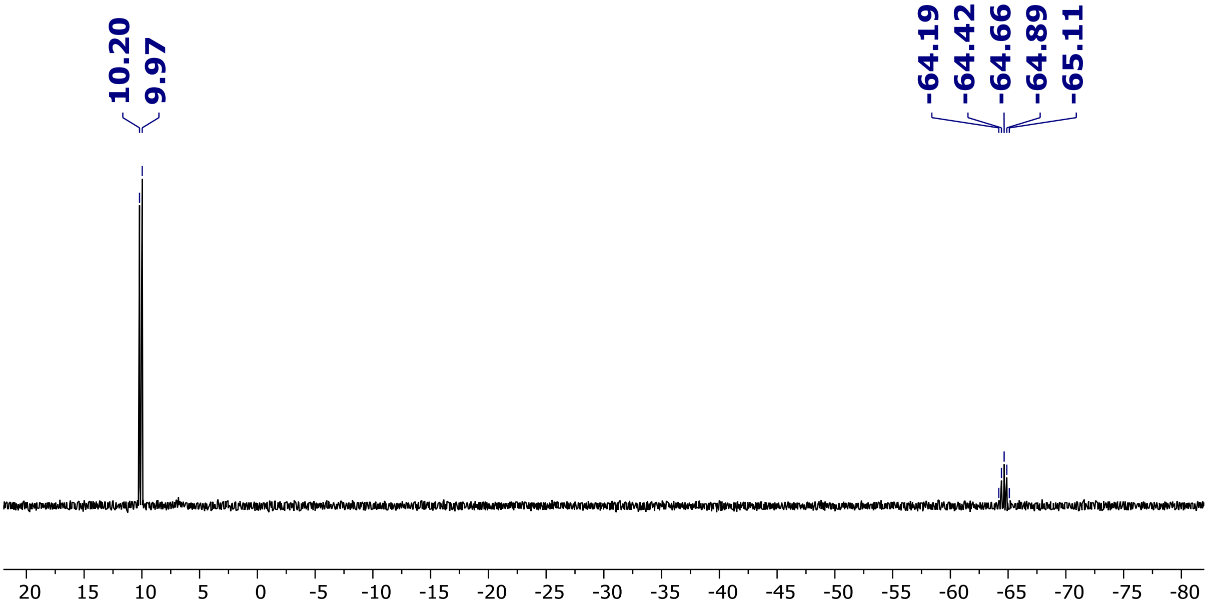


**Figure S 2**. $^{31}P\{^{1}H\}$ NMR spectrum (202 MHz, 298 K, $CD_2Cl_2$) *trans*-[Fe(dppm)$_2$(Cl)($PH_3$)][BAr$^F_4$] (**1**)

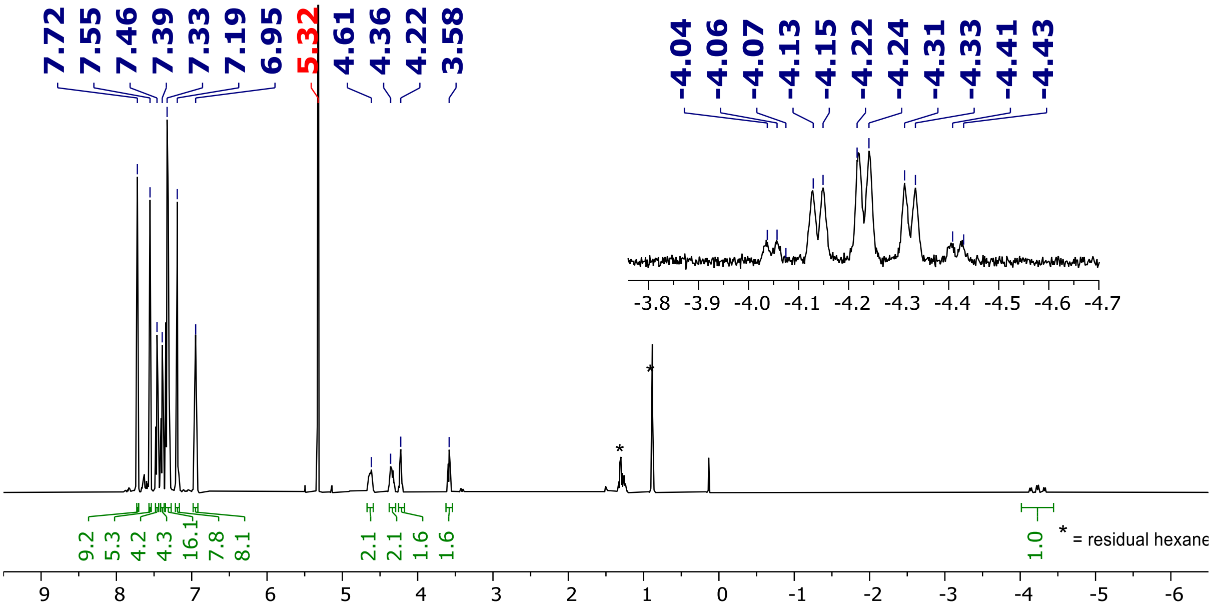


**Figure S 3.** $^{1}H$ NMR spectrum (500 MHz, 298 K, $CD_2Cl_2$) *trans*-[Fe(dppm)$_2$(H)($PH_3$)][BAr$^F$$_4$] (**2**)

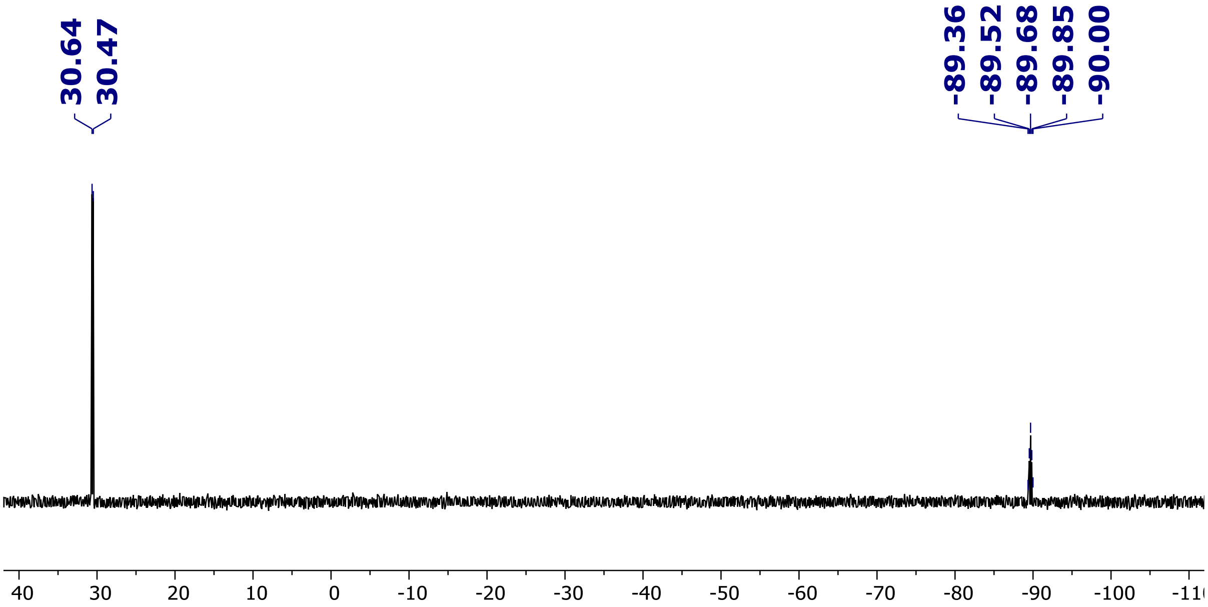


**Figure S 4.** $^{31}P\{^{1}H\}$ NMR spectrum (202 MHz, 298 K, $CD_2Cl_2$) *trans*-[Fe(dppm)$_2$(H)($PH_3$)][BAr$^F$$_4$] (**2**)

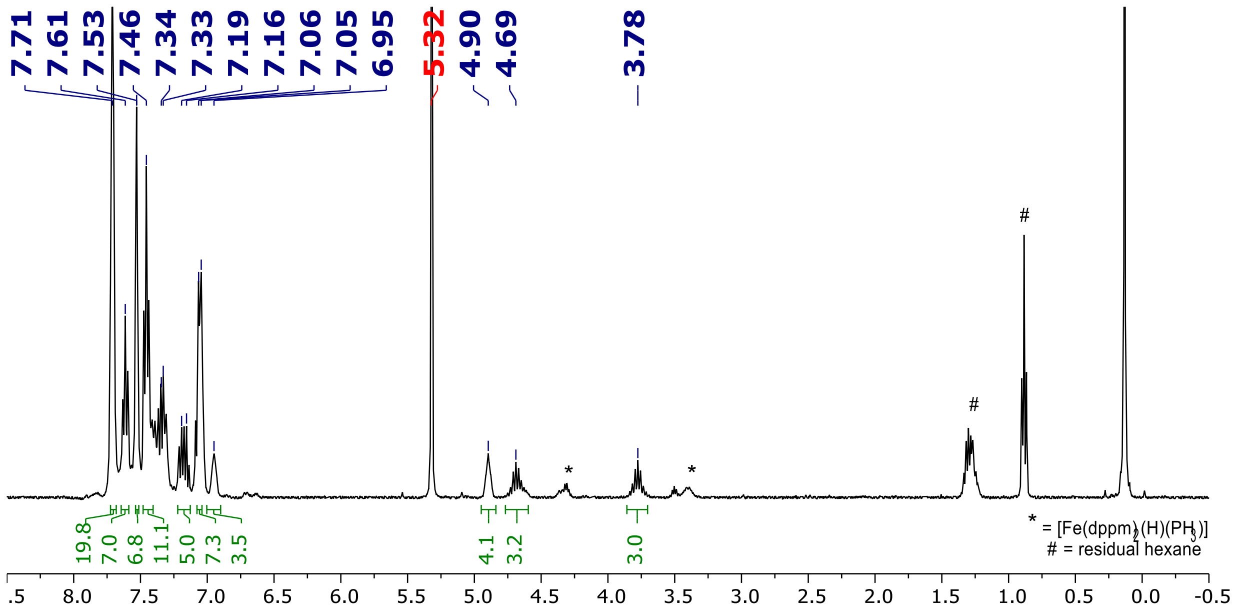


**Figure S 5.** $^1$H NMR spectrum (400 MHz, 298 K, $CD_2Cl_2$) *trans*-[Fe(dppm)$_2$($PH_3$)$_2$] 2[BAr$^F$$_4$] (**3**)

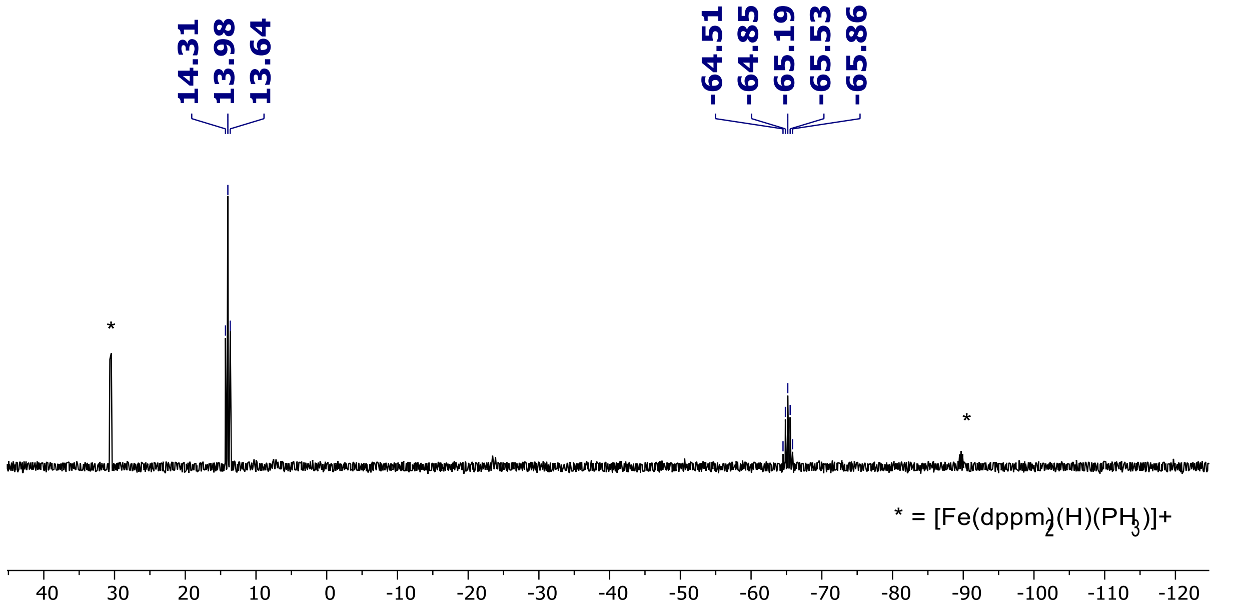


**Figure S 6.** $^{31}$P{$^1$H} NMR spectrum (162 MHz, 298 K, $CD_2Cl_2$) *trans*-[Fe(dppm)$_2$($PH_3$)$_2$] 2[BAr$^F$$_4$] (**3**)

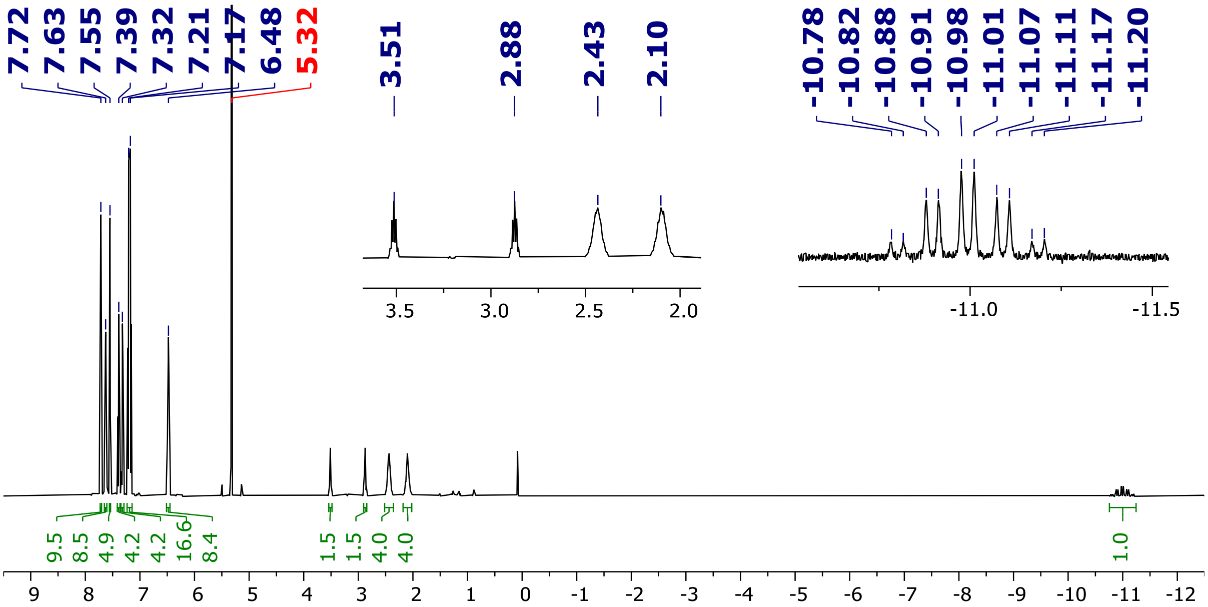


**Figure S 7.** $^{1}H$ NMR spectrum (500 MHz, 298 K, $CD_2Cl_2$) *trans*-[Fe(dppe)$_2$(H)($PH_3$)][BAr$^F$$_4$] (**4**)

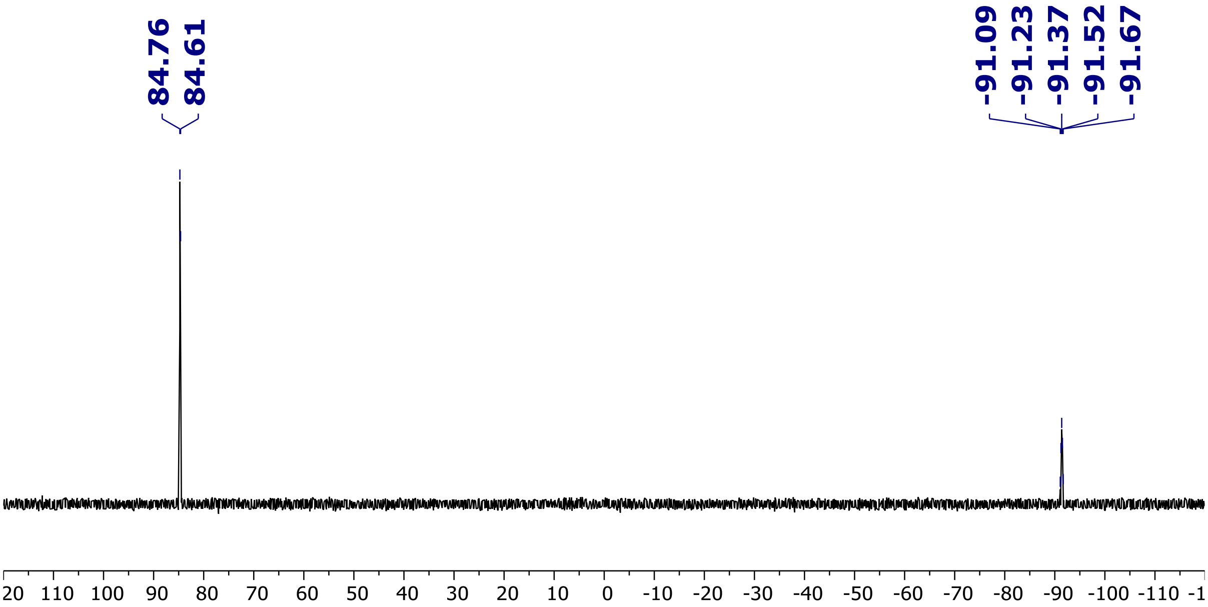


**Figure S 8.** $^{31}P\{^{1}H\}$ NMR spectrum (202 MHz, 298 K, $CD_2Cl_2$) *trans*-[Fe(dppe)$_2$(H)($PH_3$)][BAr$^F$$_4$] (**4**)

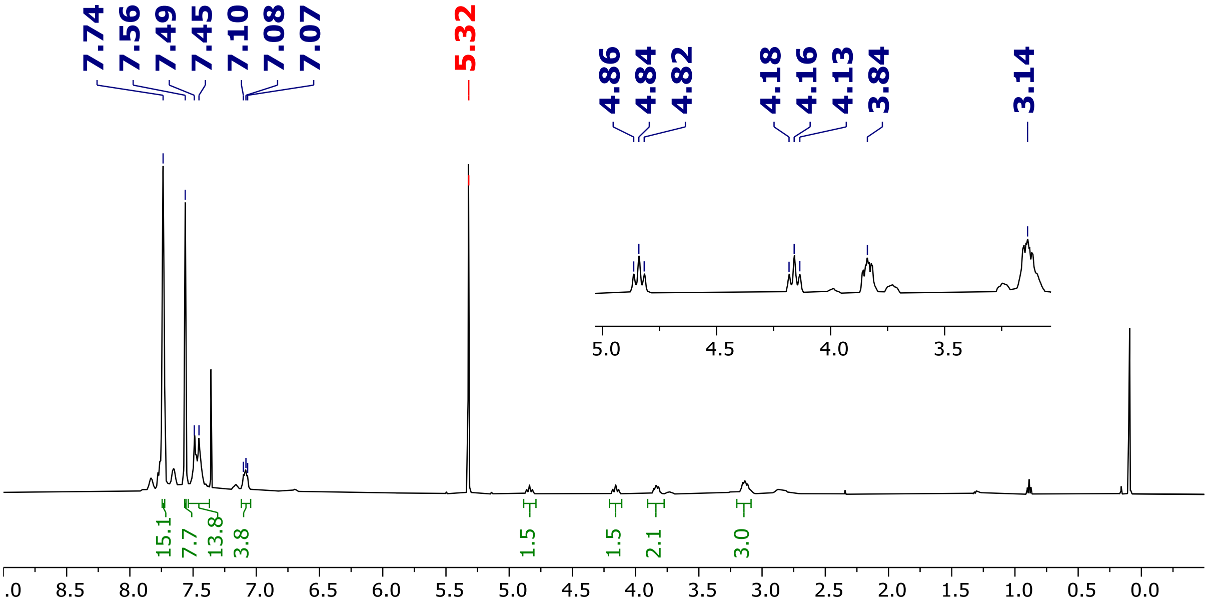


**Figure S 9.** $^{1}H$ NMR spectrum (500 MHz, 298 K, $CD_2Cl_2$) *cis*-[Fe(dppe)(Cl)($PH_3$)$_3$][$BAr^F{}_4$] (**5**)

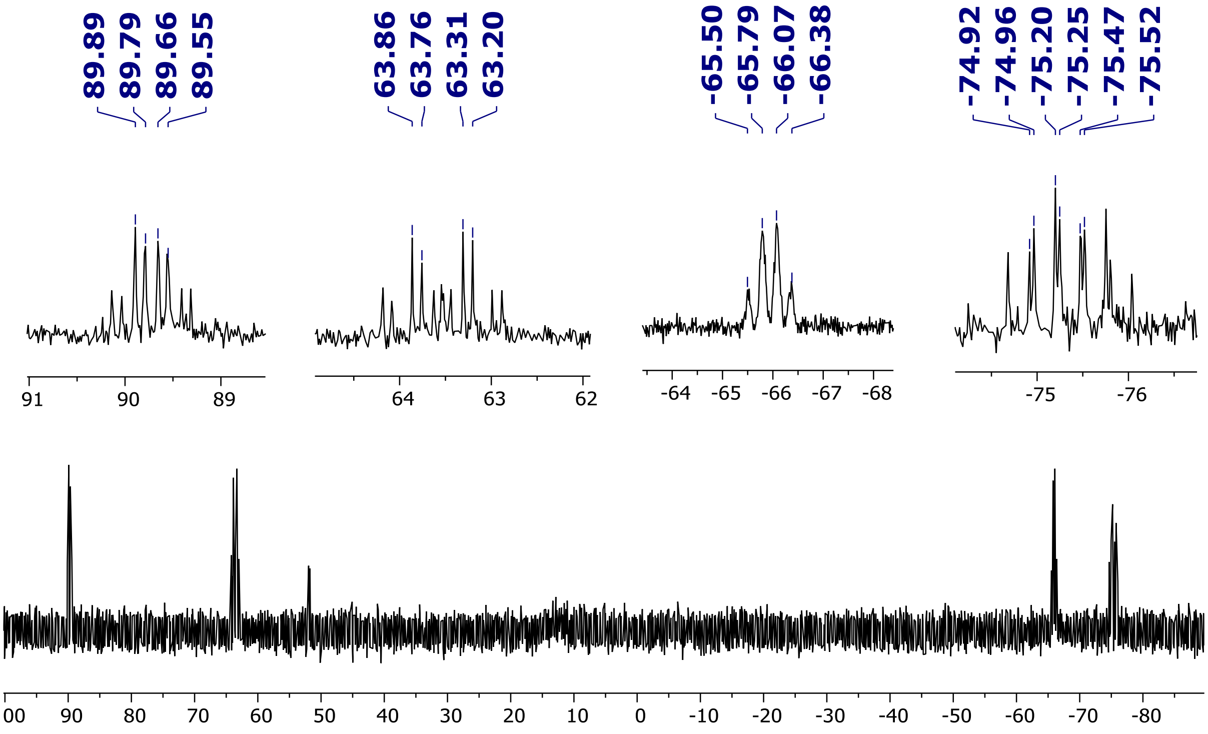


**Figure S 10.** $^{31}P\{^{1}H\}$ NMR spectrum (202 MHz, 298 K, $CD_2Cl_2$) *cis*-[Fe(dppe)(Cl)($PH_3$)$_3$][$BAr^F{}_4$] (**5**)

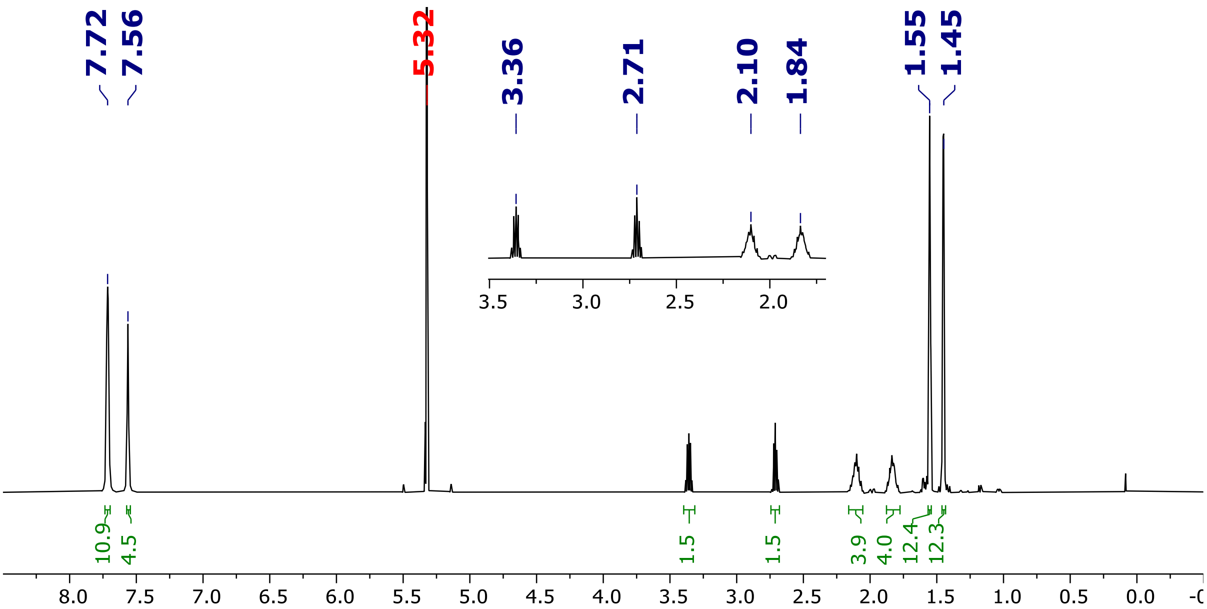


**Figure S 11.** $^{1}H$ NMR spectrum (500 MHz, 298 K, $CD_2Cl_2$) *trans*-$[Fe(dmpe)_2(Cl)(PH_3)][BAr^F_4]$ (**6**)

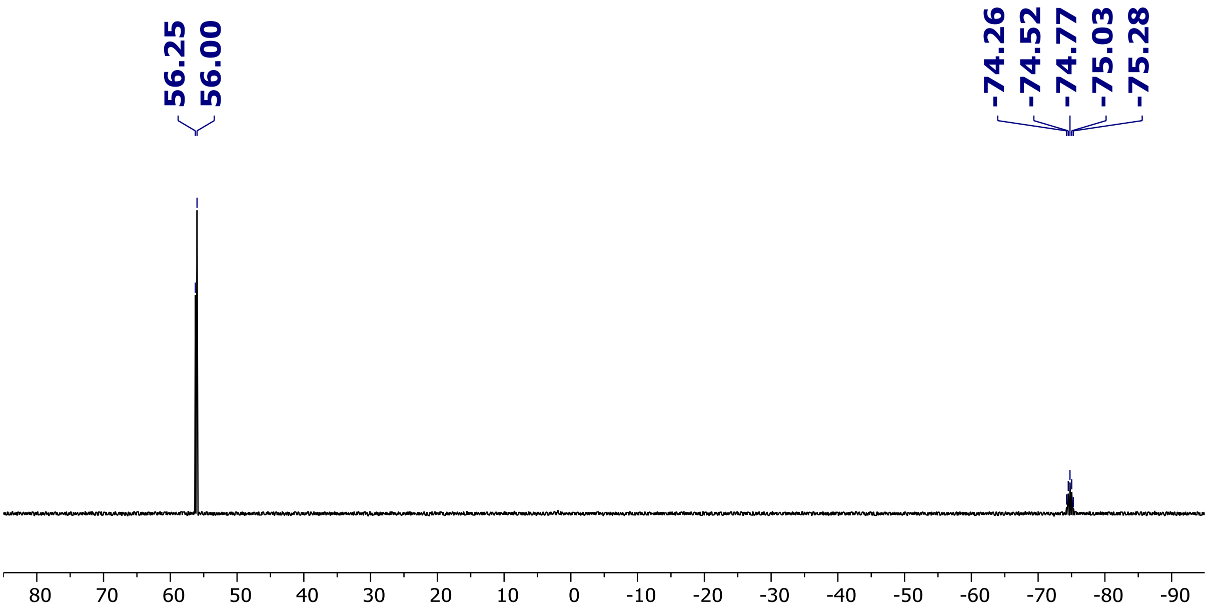


**Figure S 12.** $^{31}P\{^{1}H\}$ NMR spectrum (202 MHz, 298 K, $CD_2Cl_2$) *trans*-$[Fe(dmpe)_2(Cl)(PH_3)][BAr^F_4]$ (**6**)

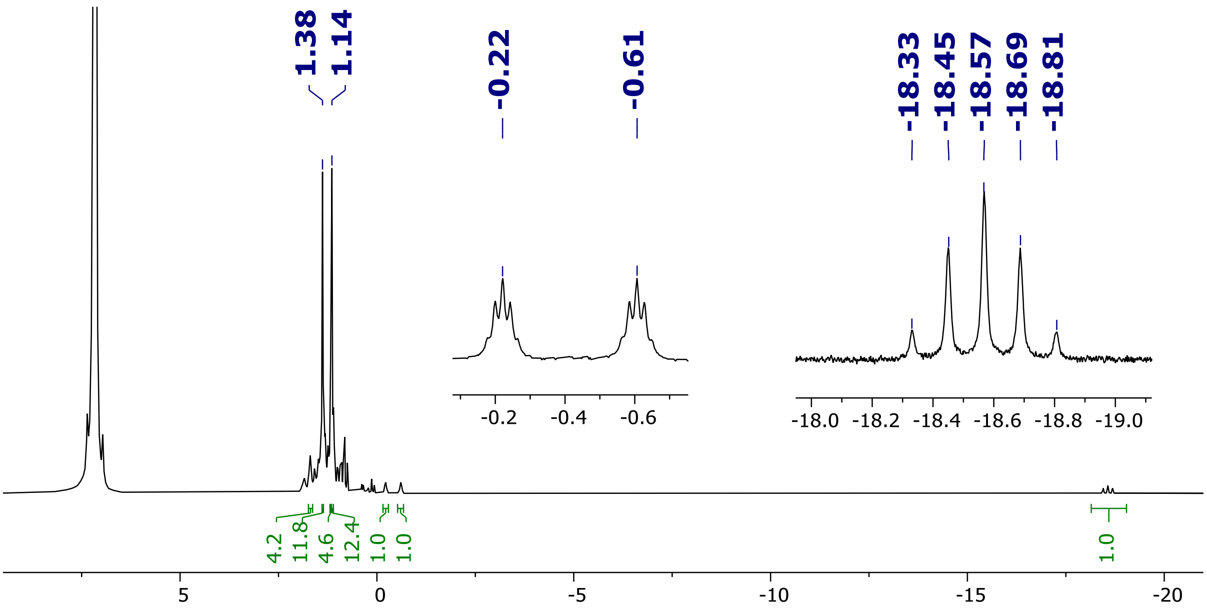


**Figure S 13.** $^{1}$H NMR spectrum (400 MHz, 298 K, $C_6D_6$) *trans*-[Fe(dmpe)$_2$(H)(PH$_2$)] (**9**)

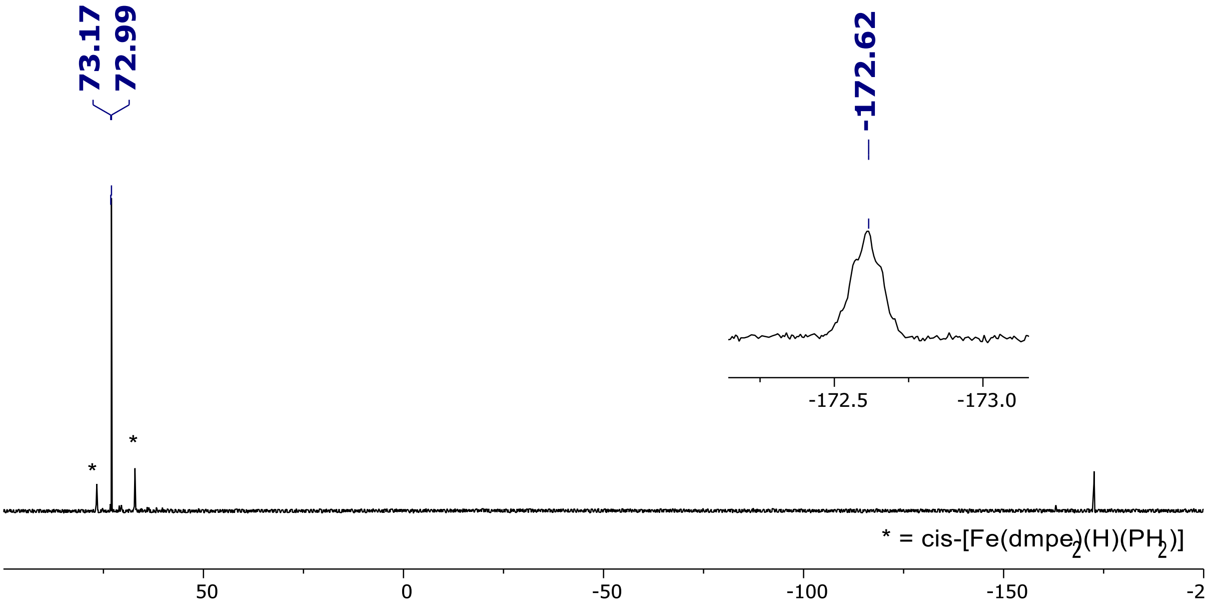


**Figure S 14.** $^{31}$P NMR spectrum (202 MHz, 298 K, $C_6D_6$) *trans*-[Fe(dmpe)$_2$(H)(PH$_2$)] (**9**)

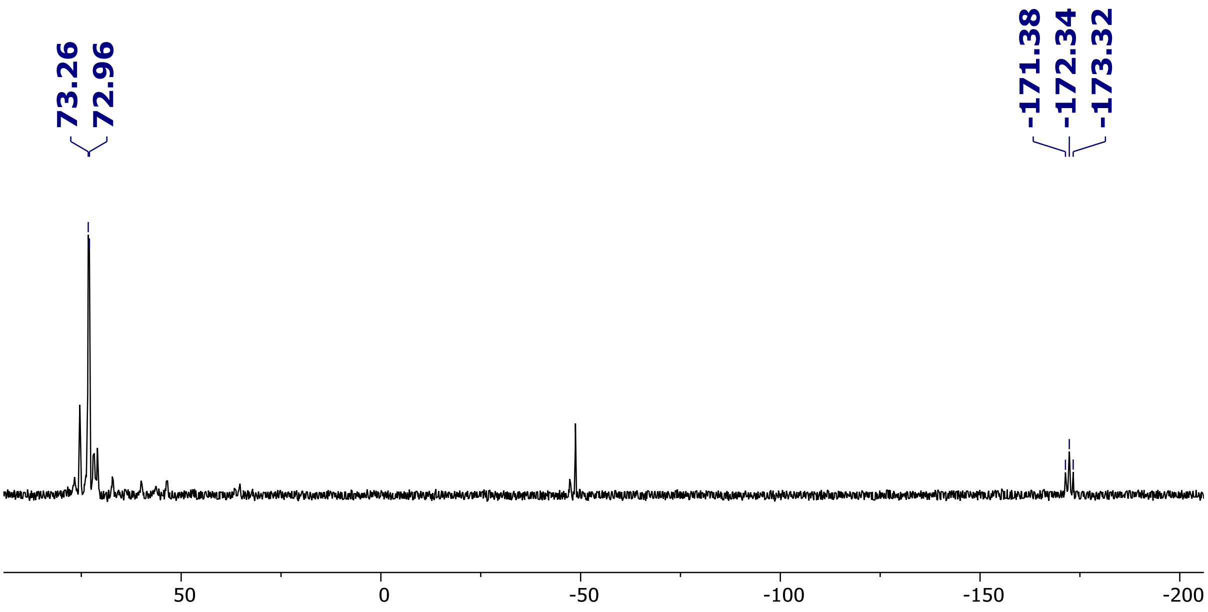


**Figure S 15.** ${}^{31}P\{{}^{1}H\}$ NMR spectrum (162 MHz, 298 K, $C_6D_6$) crude *trans*-[Fe(dmpe)$_2$(H)(PH$_2$)] (**9**), o2p = −4.00 ppm

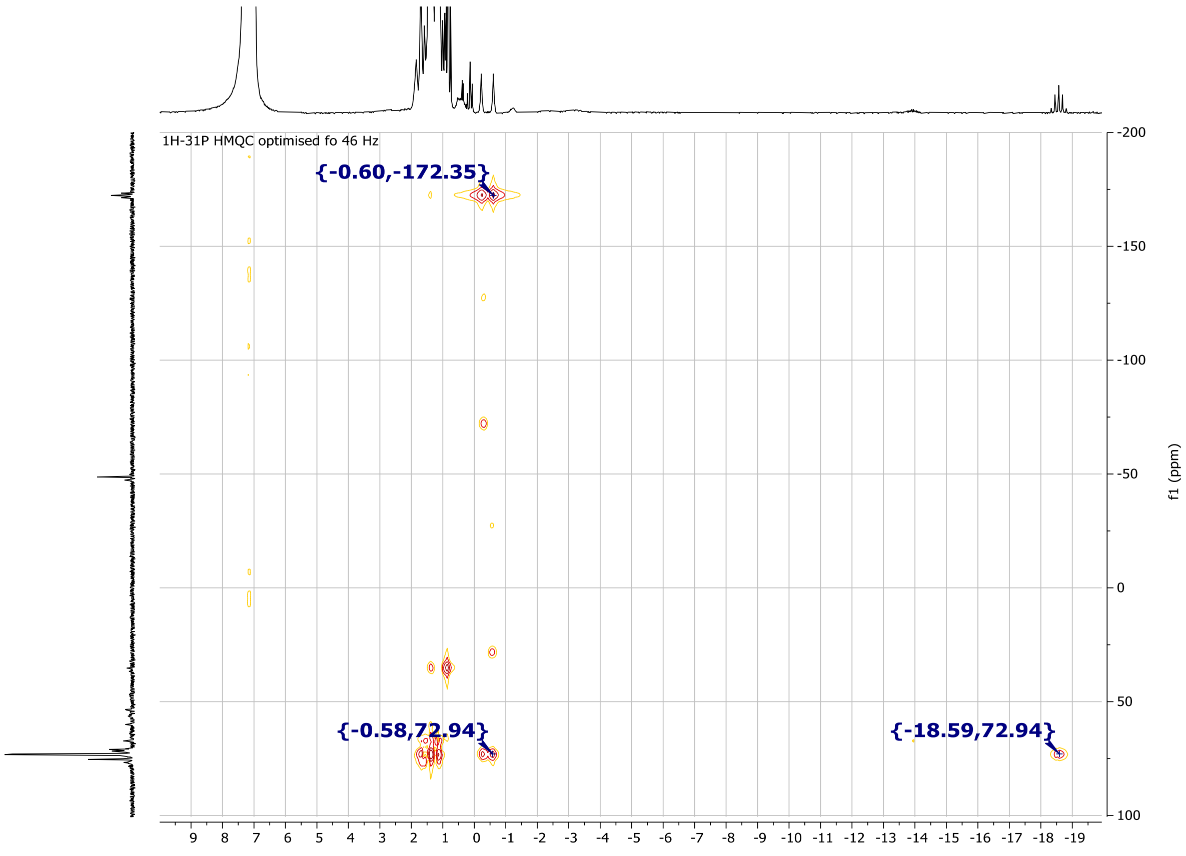


**Figure S 16.** ${}^{1}H$-${}^{31}P$ HMQC NMR spectrum (500, 202 MHz, 298 K, $C_6D_6$) (**9**)

# 6. Mass Spectroscopy

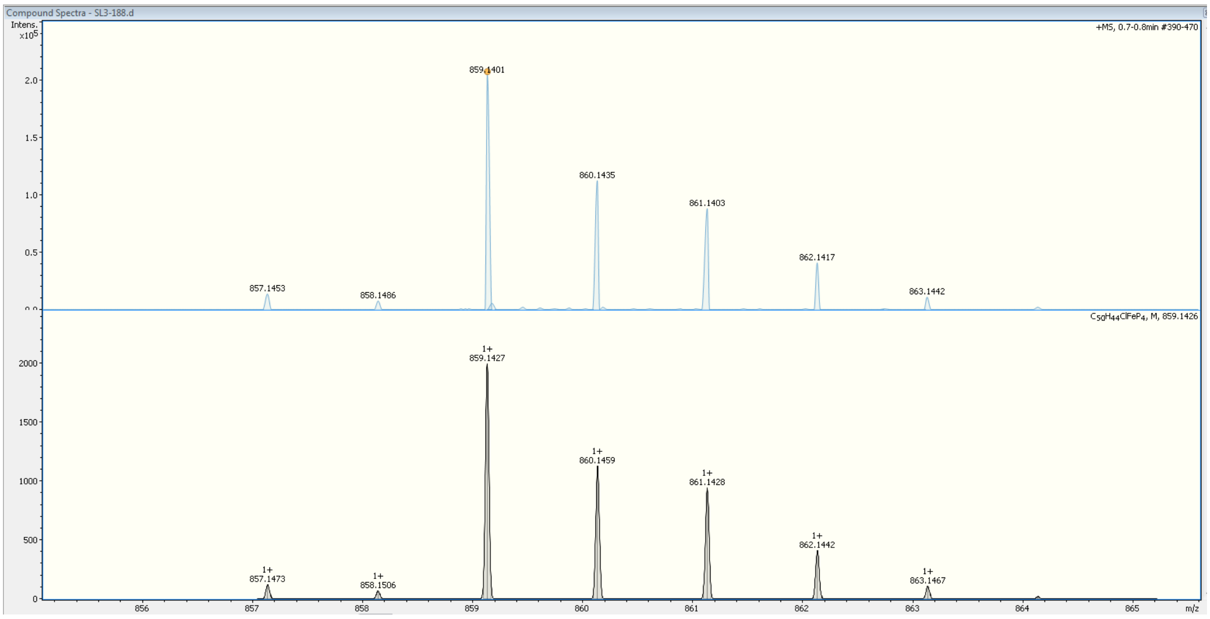


**Figure S 17.** Mass Spectrum of *trans*-[Fe(dppm)$_2$(Cl)(PH$_3$)][BAr$^F$$_4$] (**1**), measured (top) and calculated (bottom).

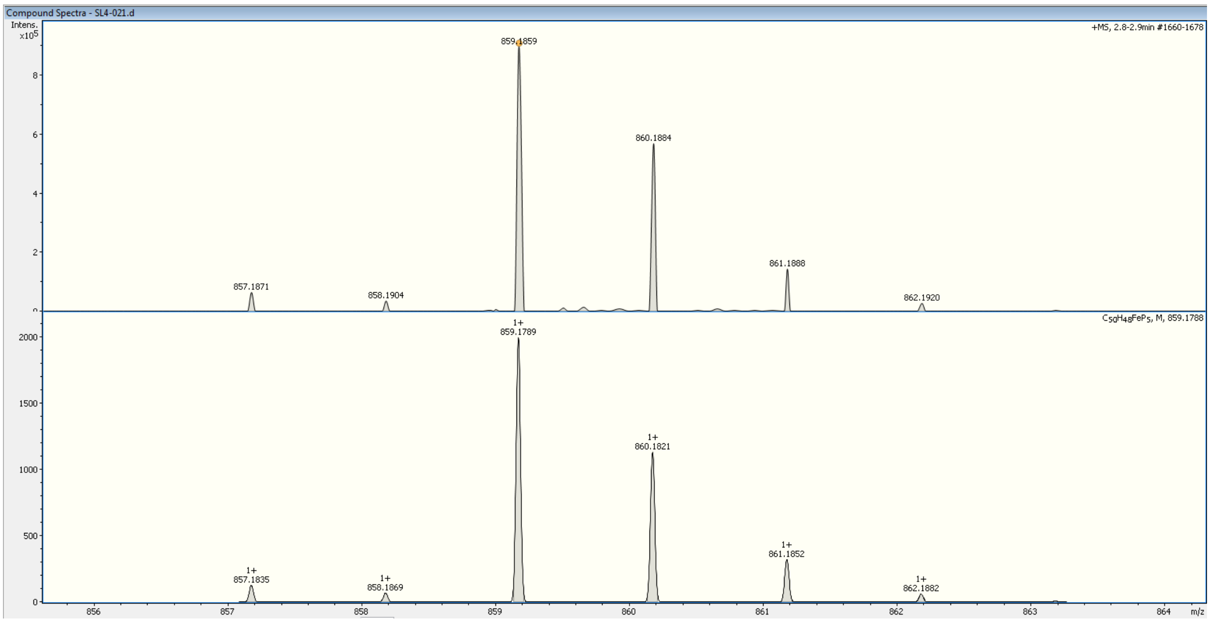


**Figure S 18**. Mass Spectrum of *trans*-[Fe(dppm)$_2$(H)(PH$_3$)][BAr$^F$$_4$] (**2**), measured (top) and calculated (bottom).

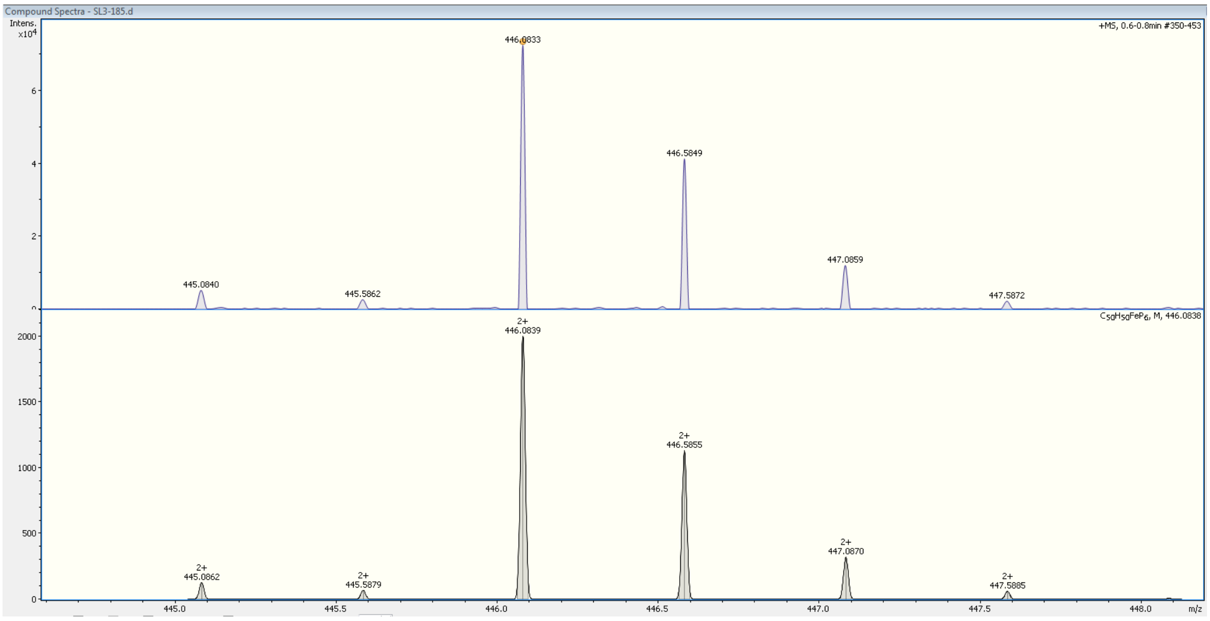


**Figure S 19**. Mass Spectrum of ) *trans*-[Fe(dppm)$_2$(PH$_3$)$_2$] 2[BAr$^F$$_4$] (**3**), measured (top) and calculated (bottom).

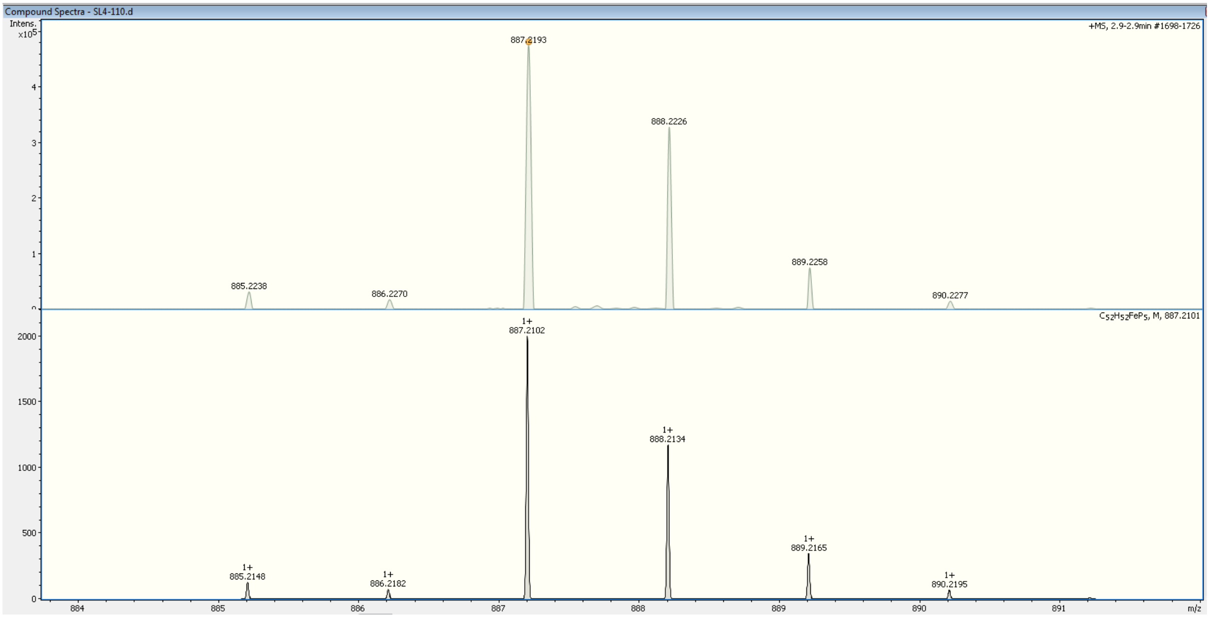


**Figure S 20**. Mass Spectrum of *trans*-[Fe(dppe)$_2$(H)(PH$_3$)][BAr$^F$$_4$] (**4**), measured (top) and calculated (bottom).

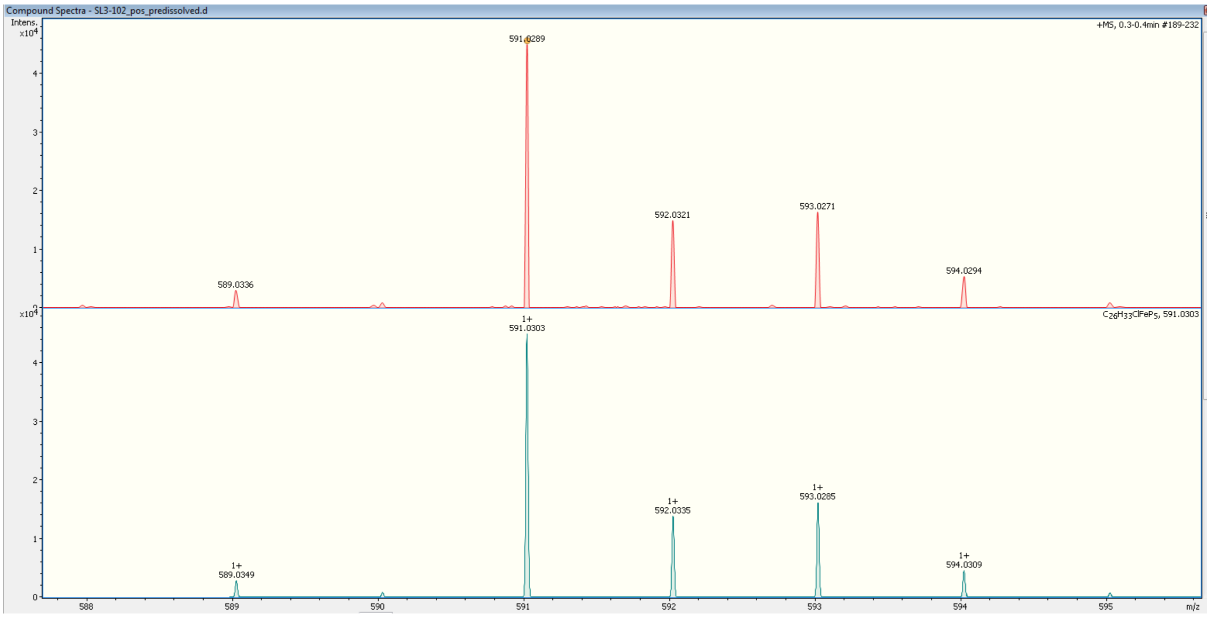


**Figure S 21**. Mass Spectrum of *cis*-[Fe(dppe)(Cl)($PH_3$)$_3$][BAr$^F{}_4$] (**5**), measured (top) and calculated (bottom).

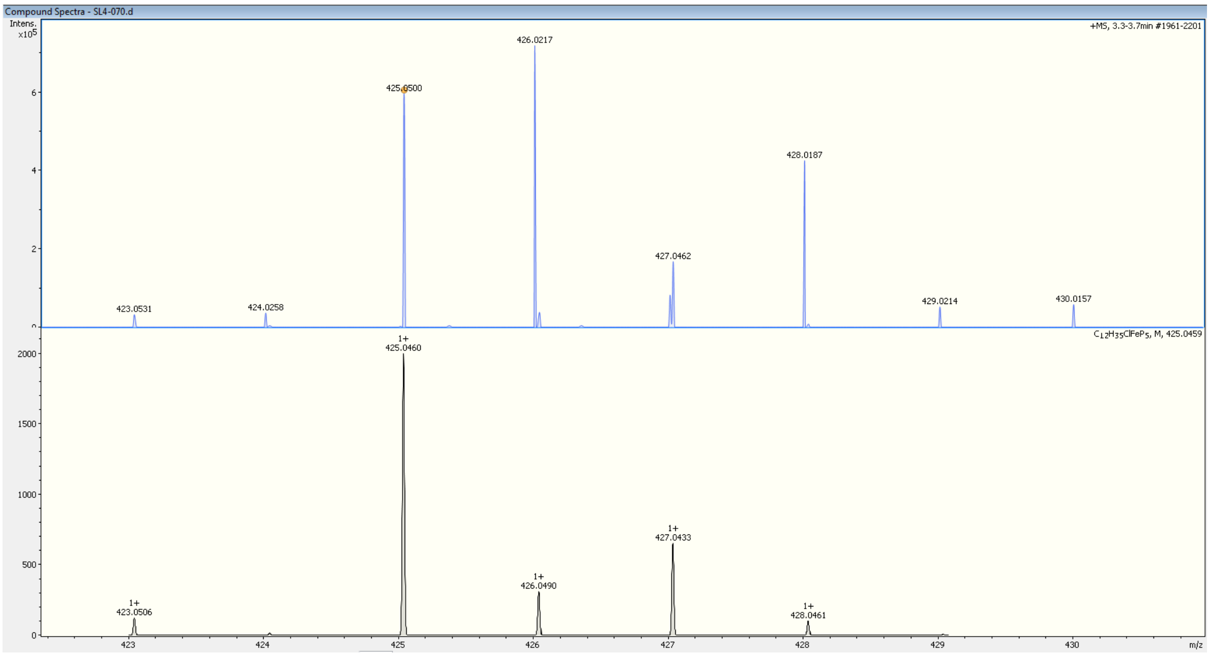


**Figure S 22.** Mass Spectrum of *trans*-[Fe(dmpe)$_2$(Cl)($PH_3$)][BAr$^F{}_4$] (**6**), measured (top) and calculated (bottom).

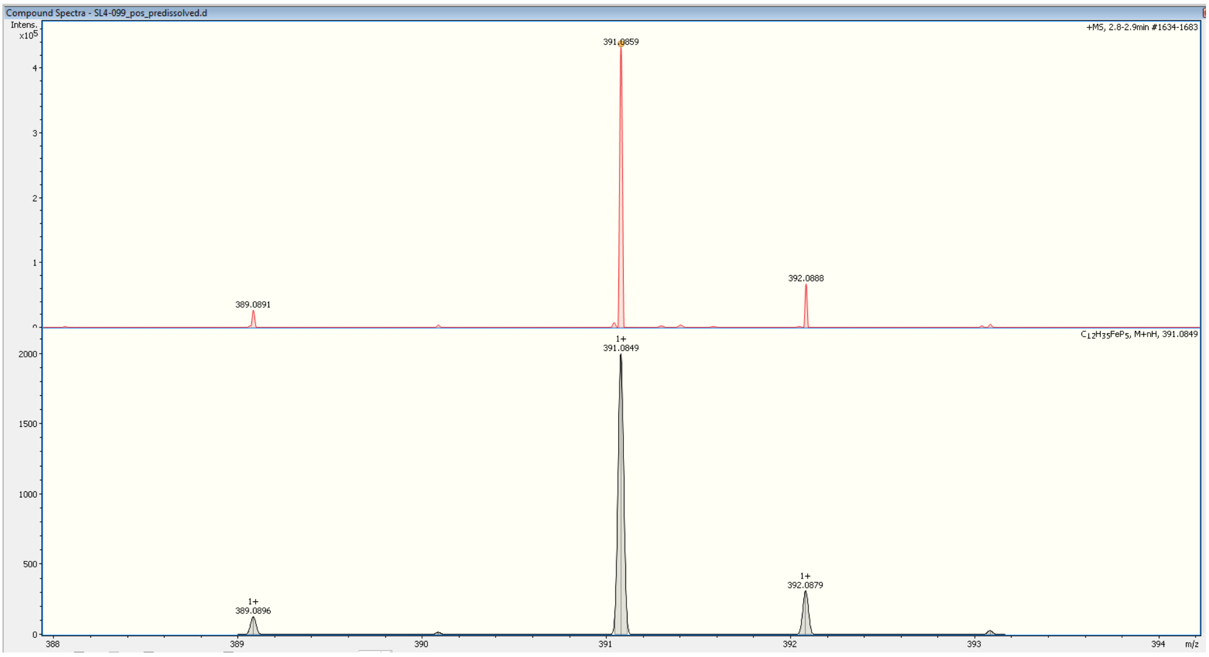


**Figure S 23**. Mass Spectrum of *trans*-[Fe(dmpe)$_2$(H)(PH$_2$)] (**9**), measured (top) and calculated (bottom).

## 7. FT-IR spectra

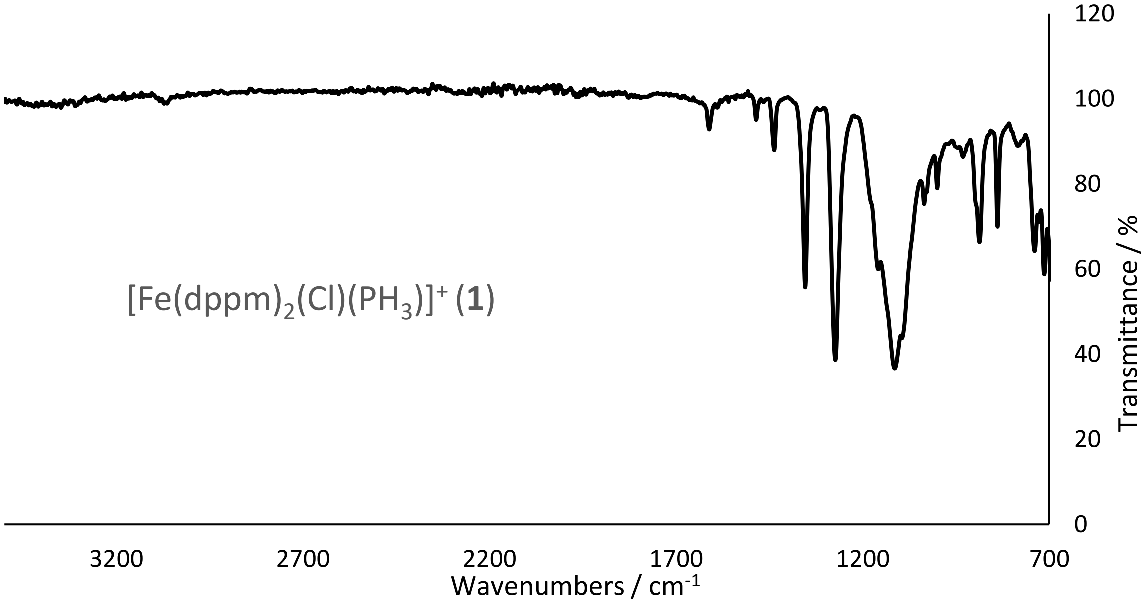

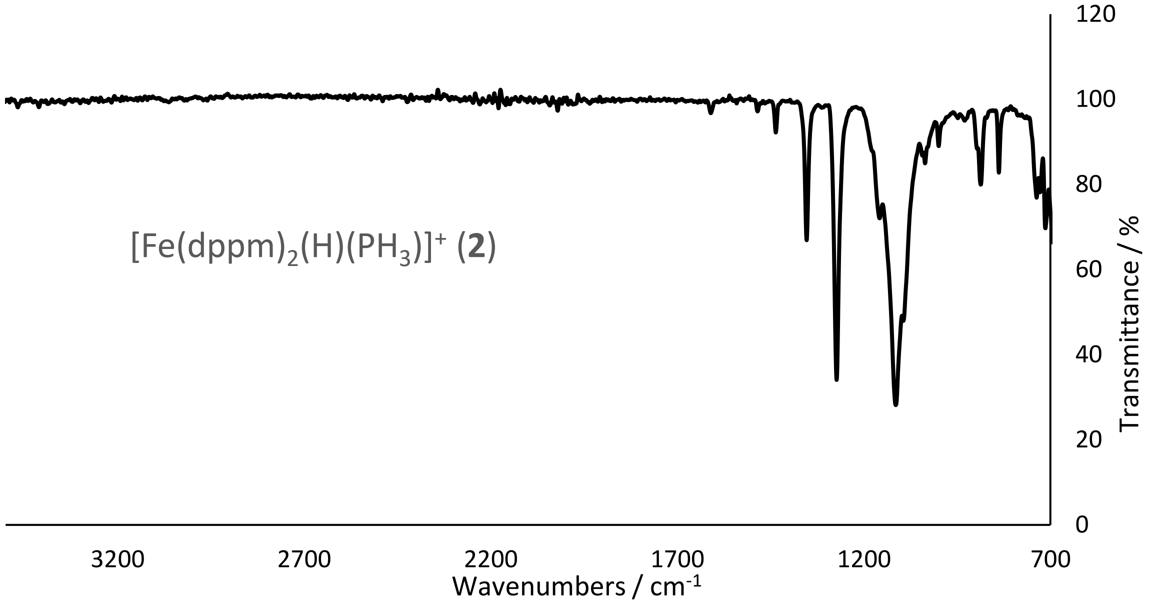
[Fe(dppm)$_2$(H)(PH$_3$)]$^+$ (**2**)
Transmittance / %
120
100
80
60
40
20
0
3200
2700
2200
1700
1200
700
Wavenumbers / cm$^{-1}$

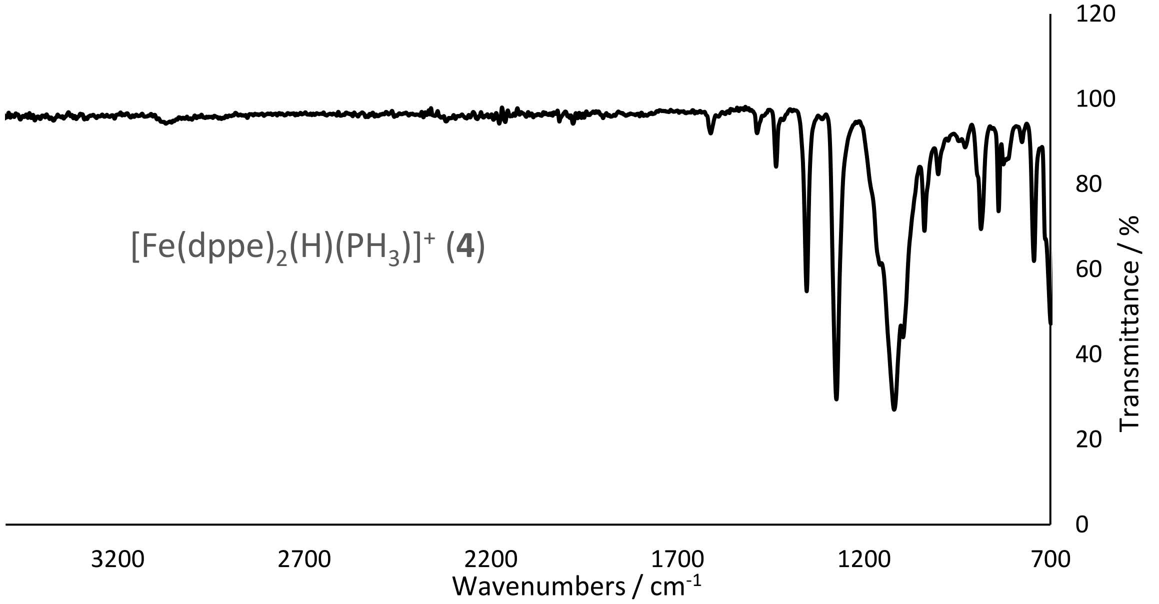
[Fe(dppe)$_2$(H)(PH$_3$)]$^+$ (**4**)
Transmittance / %
120
100
80
60
40
20
0
3200
2700
2200
1700
1200
700
Wavenumbers / cm$^{-1}$

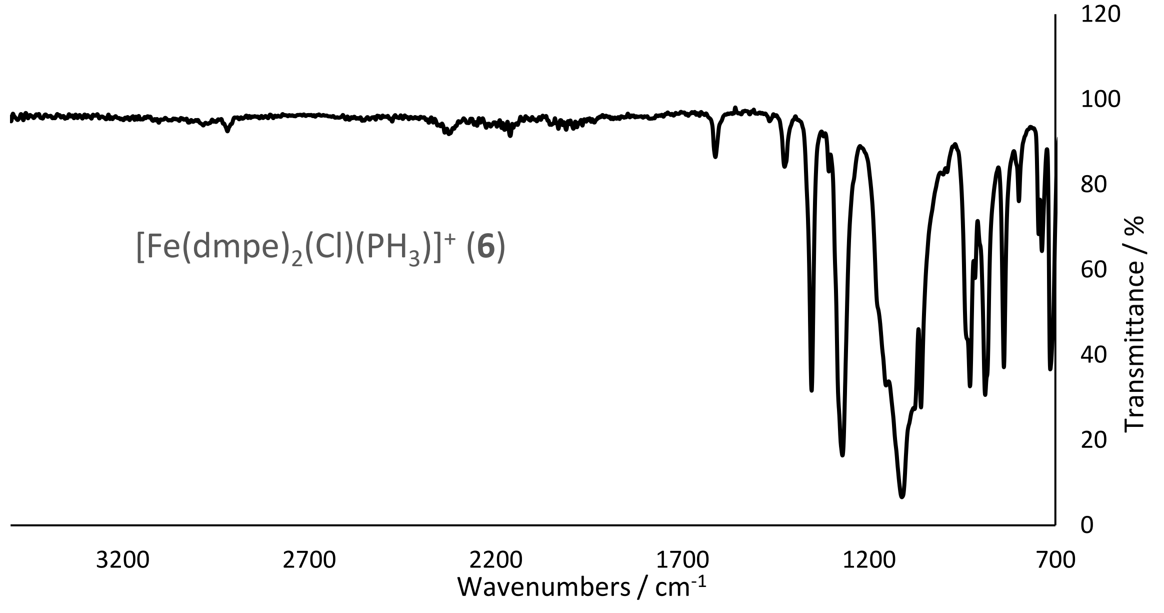
[Fe(dmpe)$_2$(Cl)(PH$_3$)]$^+$ (**6**)
120
100
80
60
40
20
0
Transmittance / %
3200
2700
2200
1700
1200
700
Wavenumbers / cm$^{-1}$

## 8. X-ray Crystallography data

See Table S1 for data.

Data were collected using an Agilent SuperNova instrument and a Cu-Kα source throughout, with the exception of those for **8**. These latter data were collected on an Agilent Xcalibur diffractometer equipped with Mo-Kα radiation. All experiments were conducted at 150 K, solved using SHELXT[6] and refined using SHELXL[7] *via* the Olex-2[8] interface. Where disorder was modelled, appropriate distance and ADP restraints were employed, on merit, to assist convergence. Additional noteworthy refinement details follow.

There is one cation, one anion, an ordered dichloromethane (90% occupancy) and a region of solvent for which the electron density is somewhat smeared in the asymmetric unit of **1**. The latter was treated *via* the solvent mask algorithm available in Olex-2 with a concomitant allowance made, in the formula as presented, for two additional $CH_2Cl_2$ moieties per unit cell. In the anion, the fluorines attached to C66 and C74 were treated for 55:45 disorder, while the entire $CF_3$ groups containing C58 and C65 were modelled to take account of 50:50 splits. The phosphorus bound hydrogens in the main feature were located and refined subject to being equidistant from P5.

In **2** there are two cations, two anions and regions of solvent in the asymmetric unit.
Cation disorder in the was confined to the phenyl ring containing C82, which was treated to take account of a 70:30 split. Promising evidence for the $PH_3$ and hydride ligands in the cations was also evident from the crystallographic analysis. In this regard, the phosphine hydrogens bonded to P5 were readily located and subsequently refined subject to being equidistant from the parent atom and equidistant from each other. Their associated $U_{iso}$ values were refined freely and these values provide some credibility to the assignments. The comparative hydrogen atoms attached to P10 were also located and similarly treated in the refinement. However, the $U_{iso}$ values for the latter reflect the fact that the distance restraints used were more necessary for the P10-bound hydrogens than those which are P5-bound. It is possible that the more diffuse nature of H10A, H10B and H10C is related to disorder of the phenyl ring that flanks the pocket in which they reside. Hydride ligands were also located, with moderate credibility, and these have been refined subject to being similar distances from the iron centres. Unsurprisingly, there was considerable disorder in the $BAr^F$ anions. In particular, the fluorines in the $CF_3$ groups based on C108, C115, C123, C155, C156 and C163 were treated for respective disorders of 60:40, 67:33, 50:50, 55:45, 50:50 and 55:45 disorders while the entire $CF_3$ moieties which included C116 and C124 were modelled to account of 65:35 and 50:50 disorder ratios, ordinally. The solvent was noted to be mostly disordered and, hence, the solvent mask algorithm in Olex-2 was used to address same. The associated calculation implied a presence of 266 electrons in a volume of 1144 $Å^3$ in three voids per unit cell. This is consistent with the presence of three molecules of dichloromethane per formula unit and an allowance has been made for same in the formula as presented.

The asymmetric unit in **3** contains half of dication, one $BAr^F$ ligand, one ordered fluorobenzene ligand and two regions of disordered solvent. The latter approximates to 1.5 molecules of fluorobenzene per unit cell, which have been accounted for in the formula as presented while the electron-density itself was addressed using the solvent mask algorithm in Olex-2. The $PH_3$ ligand hydrogens in the cation were located and refined freely, and the remainder of the cation arises by virtue of an inversion centre that is co-incident with the metal. Anion disorder was modelled for the fluorines attached to C32, C33 and C40 [75:25, 60:40 and 50:50 ratios, sequentially] while the whole of the $CF_3$ moieties containing C48 and C49 were each spread over two proximate sites in a 60:40 split.

In **4**, the asymmetric unit comprises one cation, one anion and a region of solvent. The former was entirely ordered and the hydride ligand plus the phosphorus bound hydrogens, therein, were located with ease. Hydrogens attached to P5 were refined subject to being equidistant from the parent atom while the hydride was refined without any restraints. The fluorines in the $CF_3$ groups based on C59, C60, C67, C75 and C83 were treated for successive disorders of 70:30, 50:50, 90:10, 85:15 and 85:15 while the complete $CF_3$ moieties that included C68 and C84 were modelled to account for 70:30 and 50:50 disorder ratios, respectively. The solvent electron density was very smeared and did not lend itself to ready modelling. As such, it was treated with the solvent-mask algorithm in Olex-2 and allowance made (in the formula as presented) for two molecules of hexane per asymmetric unit.
Analysis of the raw data frames for this structure suggested the presence of a small non-merohedral component that contributes approximately 3% to the diffraction. Integration of the data frames as a twin gave an $R_{int}$ for the minor component of approximately 46% and it was noted that overlapping electron-density between the two components affected almost 20% of the reflections. Refinement against a HKLF5 data set versus the integrated data that arose by ignoring the presence of the second component offered minimal improvement in the residuals. However, refinement against the deconvoluted intensities arising from the major component (from a twin integration) did afford a substantial attenuation of both the residuals and the metric-data esds, relative to the aforementioned approaches. As such, the results from this optimal refinement are presented herein.

The asymmetric unit in **5** contains one cation and one anion. The hydrogen atoms in the $PH_3$ ligands of the former were readily located and refined (with free $U_{iso}$ values) subject to being same distance from the relevant pnictogen. The fluorines in the $CF_3$ groups based on C33, C34, C41, C49 and C50 were treated for respective disorders of 65:35, 60:40, 50:50, 50:50 and 65:35 while those attached to C57 were each modelled as three-component split in a 40:30:30 ratio.

There is one cation and one anion in the asymmetric unit of **6**. In order to extract maximum information from the difference Fourier electron-density map (particularly in the cation region), extensive modelling was undertaken for the three disordered areas in the anion. Thus, the fluorines attached to C19 were modelled for a 50:23:17 split while the entire $CF_3$ groups that include C20 and C44 were each treated as three parts in respective fractions ratios of 45:31:24 and 63:24:13. Unfortunately, these cardinal efforts did not result in the emergence of any identifiable hydrogen positions associated with the atom

assigned as P5 in the cation. Of course, the putative phosphine could be disordered with the *trans* chloride ligand and, were this the case, it might account for the inability to locate the P5-bound hydrogens. However, the Fe1-P5 distance herein (2.2176(6) Å) bears more similarity to the Fe-P distances noted for structure **2** [2.1973(10)/2.1989(11)Å] than the Fe1-Cl1 of 2.2758(6) Å. Swapping the assignment of Cl1 and P5 in **6** resulted in a less satisfactory convergence, evidenced by and *R*1 values of 4.14% and a *w*R2 of 11.72%. Assignment of P5 as a chloride ligand rendered an *R*1 of 3.92% and a *wR*2 of 10.76% which, relative to the values from the model as presented [3.90/10.67] do not provide unambiguous crystallographic evidence for the P5 assignment. However, the spectroscopic results were unequivocal with respect to characterisation of this material, and the final least squares was implemented on the basis that the cation possesses one phosphine and one chloride which were not hostages to disorder.

Half of a dication, one $BAr^F$ anion and four molecules of fluorobenzene constitute the asymmetric unit in **7**. The remainder of the cation arises *via* crystallographic inversion symmetry. The fluorines in the $CF_3$ groups based on C82, C83, C90, C91 and C99 were treated for respective disorder ratios of 85:15, 70:30, 50:50, 50:50 and 75:25.
The solvent, though identifiable, was disordered and it was ultimately treated using the solvent mask algorithm in Olex-2. Allowance has been made for same in the formula as presented.

The asymmetric unit in **8** comprises two cations, two anions and two regions of solvent. While the cationic complexes were entirely ordered, some $CF_3$ moieties in the $BAr^F$ anions were hostage to disorder. In particular, the fluorines in attached to C111, C119, C127, C143 and C157 were treated for respective disorder ratios of 75:25, 50:50, 75:25, 75:25 and 70:30. The solvent, while recognisable in one area, was ultimately treated using the solvent mask algorithm in Olex-2 and an allowance has been made for the presence of four molecules of dichloromethane per unit cell in the formula as presented, as a consequence of the masking analysis.

In **9,** the asymmetric unit comprises two molecules. All carbons present were found to be disordered in a 50:50 ratio and were refined subject to chemically similar distances being equal to each other. The hydrides, H1 and H2, were located readily and refined without restraints other than being equidistant from the relevant iron centre to which each is attached. The hydrogens attached to P5 and P10 included in the model were derived from residual electron density. The P-H distances were refined subject to being similar to each other as were the H…H distances. However, while the certainty in the locations of the hydrides is high, the certainty in the phosphorus-bound hydrogen positions is not at the same level. This is reflected in the $U_{iso}$ values associated with H1-H6. Additionally, the electron density suggested that the $PH_2$ group hydrogen atoms might well be disordered over two positions but no effort was made to refine this. The crystal sample was also twinned and the raw data were integrated to take account of this twinning about the *c* axis. Given the level of disorder, the structure was also solved and refined in space group *P*1 and the outcome of this exercise was to confirm that optimal solution and refinement, as presented here, is in the correctly assigned space group (i.e. *P*-1).

Crystallographic data for all compounds have been deposited with the Cambridge Crystallographic Data Centre as supplementary publications CCDC 2329812-2329820 for complexes **1** to **9** respectively. Copies of these data can be obtained free of charge on application to CCDC, 12 Union Road, Cambridge CB2 1EZ, UK [fax(+44) 1223 336033, e-mail: deposit@ccdc.cam.ac.uk.

**TABLE S1**

| Identification code | **Complex 1** | **Complex 2** | **Complex 3** | **Complex 4** | **Complex 5** |
|---|---|---|---|---|---|
| Empirical formula | $C_{83.9}H_{62.8}BCl_{4.8}F_{24}FeP_5$ | $C_{167}H_{126}B_2F_{48}Fe_2P_{10}Cl_3$ | $C_{141}H_{99}B_2F_{53}FeP_6$ | $C_{96}H_{92}BF_{24}FeP_5$ | $C_{58}H_{45}BClF_{24}FeP_5$ |
| Formula weight | 1918.61 | 3594.04 | 3063.49 | 1923.20 | 1454.90 |
| Crystal system | triclinic | triclinic | triclinic | triclinic | triclinic |
| Space group | *P*−1 | *P*−1 | *P*−1 | *P*−1 | *P*−1 |
| *a*/ Å | 14.4800(4) | 18.5216(4) | 12.9511(4) | 12.0695(3) | 13.2525(4) |
| *b*/ Å | 15.3039(5) | 18.8792(5) | 16.6060(5) | 19.3456(5) | 14.6498(4) |
| *c*/ Å | 20.3503(6) | 24.6526(6) | 18.6304(4) | 20.0793(5) | 16.8163(3) |
| $\alpha$/ ° | 105.629(3) | 90.772(2) | 115.638(3) | 76.821(2) | 103.751(2) |
| $\beta$/ ° | 98.253(2) | 90.527(2) | 105.764(2) | 82.312(2) | 93.629(2) |
| $\gamma$/ ° | 91.535(2) | 91.118(2) | 91.048(3) | 79.944(2) | 91.988(2) |
| $U$/ $Å^3$ | 4287.8(2) | 8617.5(4) | 3433.55(19) | 4473.1(2) | 3160.72(14) |
| *Z* | 2 | 2 | 1 | 2 | 2 |
| $\rho_{calc}$/ g $cm^{-3}$ | 1.486 | 1.385 | 1.482 | 1.428 | 1.529 |
| $\mu$/ $mm^{-1}$ | 4.521 | 3.542 | 2.636 | 3.044 | 4.485 |
| *F*(000) | 1940.0 | 3646.0 | 1548.0 | 1980.0 | 1464.0 |
| Crystal size/ $mm^3$ | 0.244 × 0.112 × 0.083 | 0.117 × 0.096 × 0.071 | 0.204 × 0.148 × 0.073 | 0.22 × 0.079 × 0.061 | 0.111 × 0.106 × 0.028 |
| 2θ range for data collection/° | 8.244 to 146.124 | 6.752 to 146.442 | 7.18 to 146.53 | 7.226 to 140.14 | 6.692 to 146.61 |
| Index ranges | $-17 \le h \le 17$, $-18 \le k \le 18$, $-25 \le l \le 25$ | $-22 \le h \le 22$, $-21 \le k \le 23$, $-30 \le l \le 21$ | $-16 \le h \le 15$, $-20 \le k \le 19$, $-23 \le l \le 21$ | $-14 \le h \le 14$, $-23 \le k \le 23$, $-24 \le l \le 23$ | $-16 \le h \le 14$, $-17 \le k \le 18$, $-20 \le l \le 17$ |
| Reflections collected | 56056 | 69192 | 31319 | 57778 | 31126 |
| Independent reflections, $R_{int}$ | 16967, 0.0417 | 33819, 0.0419 | 13533, 0.0289 | 16771, 0.0447 | 12540, 0.0452 |
| Data/restraints/parameters | 16967/275/1180 | 33819/809/2325 | 13533/278/928 | 16771/429/1253 | 12540/519/1036 |
| Goodness–of–fit on $F^2$ | 1.048 | 1.025 | 1.059 | 0.895 | 1.020 |
| Final *R*1, *wR*2 [$I$>=2σ($I$)] | 0.0491, 0.1302 | 0.0625, 0.1529 | 0.0527, 0.1439 | 0.0408, 0.0975 | 0.0554, 0.1345 |
| Final *R*1, *wR*2 [all data] | 0.0591, 0.1382 | 0.0864, 0.1682 | 0.0622, 0.1519 | 0.0564, 0.1012 | 0.0768, 0.1481 |
| Largest diff. peak/hole/ e $Å^{-3}$ | 0.66/−0.60 | 0.95/−0.78 | 0.79/−0.62 | 0.74/−0.44 | 0.84/−0.64 |

**TABLE S1 continued**

| Identification code | **Complex 6** | **Complex 7** | **Complex 8** | **Complex 9** |
|---|---|---|---|---|
| Empirical formula | $C_{44}H_{47}BClF_{24}FeP_5$ | $C_{131}H_{98}BCl_6F_{28}Fe_{3.5}P_6$ | $C_{170}H_{124}B_2Cl_6F_{48}Fe_2P_8$ | $C_{12}H_{35}FeP_5$ |
| Formula weight | 1288.77 | 2808.89 | 3672.46 | 390.10 |
| Crystal system | orthorhombic | triclinic | triclinic | triclinic |
| Space group | *Pbca* | *P*−1 | *P*−1 | *P*−1 |
| $a$/ Å | 19.2081(2) | 18.7126(3) | 12.8335(3) | 9.2246(3) |
| $b$/ Å | 22.6470(2) | 19.0429(3) | 19.4549(5) | 12.4638(4) |
| $c$/ Å | 24.3334(2) | 20.9298(3) | 34.4336(9) | 17.3198(7) |
| $\alpha$/ ° | 90 | 110.015(1) | 103.190(2) | 89.872(3) |
| $\beta$/ ° | 90 | 107.936(1) | 95.5403(19) | 88.482(3) |
| $\gamma$/ ° | 90 | 98.656(1) | 98.602(2) | 89.228(3) |
| $U$/ Å$^3$ | 10585.17(17) | 6388.13(18) | 8199.6(4) | 1990.44(12) |
| $Z$ | 8 | 2 | 2 | 4 |
| $\rho_{calc}$/ g cm$^{-3}$ | 1.617 | 1.460 | 1.487 | 1.302 |
| $\mu$/ mm$^{-1}$ | 5.262 | 5.778 | 0.460 | 9.747 |
| $F$(000) | 5200.0 | 2848.0 | 3720.0 | 832.0 |
| Crystal size/ mm$^3$ | 0.211 × 0.141 × 0.044 | 0.251 × 0.142 × 0.108 | 0.260 × 0.233 × 0.119 | 0.199 × 0.167 × 0.083 |
| 2θ range for data collection/° | 7.044 to 146.978 | 7.224 to 145.864 | 5.68 to 54.968 | 7.092 to 146.604 |
| Index ranges | −23 ≤ h ≤ 23, −20 ≤ k ≤ 27, −29 ≤ l ≤ 30 | −23 ≤ h ≤ 14, −23 ≤ k ≤ 23, −20 ≤ l ≤ 25 | −10 ≤ h ≤ 16, −25 ≤ k ≤ 25, −44 ≤ l ≤ 43 | −11 ≤ h ≤ 11, −15 ≤ k ≤ 15, −12 ≤ l ≤ 21 |
| Reflections collected | 148325 | 72579 | 82130 | 12412 |
| Independent reflections, $R_{int}$ | 10606, 0.0609 | 25058, 0.0273 | 37413, 0.0472 | 12412, * |
| Data/restraints/parameters | 10606/684/867 | 25058/330/1465 | 37413/330/2206 | 12412/652/598 |
| Goodness–of–fit on F$^2$ | 1.032 | 1.048 | 1.020 | 0.870 |
| Final $R$1, $wR$2 [$I$>=2σ($I$)] | $R_1$ = 0.0390, 0.1026 | $R_1$ = 0.0400, 0.1048 | $R_1$ = 0.0599, 0.1177 | $R_1$ = 0.0321, 0.0683 |
| Final $R$1, $wR$2 [all data] | $R_1$ = 0.0443, 0.1067 | $R_1$ = 0.0490, 0.1099 | $R_1$ = 0.1037, 0.1386 | $R_1$ = 0.0458, 0.0712 |
| Largest diff. peak/hole/ e Å$^{-3}$ | 0.41/−0.41 | 0.54/−0.51 | 0.54/−0.51 | 0.42/−0.28 |

*HKLF 5 file, $R_{int}$ not applicable

## 9. References


1. Martínez-Martínez, A. J.; Weller, A. S., Solvent-free anhydrous Li+, Na+ and K+ salts of [B(3,5-(CF3)2C6H3)4]−, [BArF4]−. Improved synthesis and solid-state structures. *Dalton Transactions* **2019,** *48* (11), 3551-3554.
2. Aresta, M.; Giannoccaro, P.; Rossi, M.; Sacco, A., Hidrido-complexes of iron(IV) and iron(II). *Inorganica Chimica Acta* **1971,** *5*, 115-118.
3. Hills, A.; Hughes, D. L.; Jimenez-Tenorio, M.; Leigh, G. J.; Rowley, A. T., Bis[1,2-bis(dimethylphosphino)ethane]dihydrogenhydridoiron(II) tetraphenylborate as a model for the function of nitrogenases. *Journal of the Chemical Society, Dalton Transactions* **1993,** (20), 3041-3049.
4. Doyle, L. R.; Hill, P. J.; Wildgoose, G. G.; Ashley, A. E., Teaching old compounds new tricks: efficient N2 fixation by simple Fe(N2)(diphosphine)2 complexes. *Dalton Transactions* **2016,** *45* (18), 7550-7554.
5. Barber, T.; Argent, S. P.; Ball, L. T., Expanding Ligand Space: Preparation, Characterization, and Synthetic Applications of Air-Stable, Odorless Di-tert-alkylphosphine Surrogates. *ACS Catalysis* **2020,** *10* (10), 5454-5461.
6. Sheldrick, G., SHELXT - Integrated space-group and crystal-structure determination. *Acta Crystallographica Section A* **2015,** *71* (1), 3-8.
7. Sheldrick, G., Crystal structure refinement with SHELXL. *Acta Crystallographica Section C* **2015,** *71* (1), 3-8.
8. Dolomanov, O. V.; Bourhis, L. J.; Gildea, R. J.; Howard, J. A. K.; Puschmann, H., OLEX2: a complete structure solution, refinement and analysis program. *Journal of Applied Crystallography* **2009,** *42* (2), 339-341.
